# Chloride Binding in Trimeric Coiled Coils: Free Energy and Structural Determinants from Molecular Simulations

**DOI:** 10.1021/acs.jcim.5c02565

**Published:** 2026-01-30

**Authors:** Riccardo Nifosì, Luca Bellucci

**Affiliations:** Istituto Nanoscienze, Consiglio Nazionale delle Ricerche (CNR-NANO), and Scuola Normale Superiore, Piazza San Silvestro 12, 56127 Pisa, Italy

## Abstract

Coiled coils, owing to their simple yet versatile architecture, serve as valuable model systems for both experimental and computational studies in protein science. Whereas the sequence–structure relationships that govern their oligomeric state and stability have been thoroughly investigated, important gaps remain, most notably regarding the role of central chloride ions coordinated by asparagine triads observed in several trimeric coiled-coil (TCC) crystal structures. To investigate the thermodynamics of chloride binding at this site, we performed extensive molecular simulations using metadynamics and alchemical free-energy calculations, both enhanced with replica exchange, to determine the chloride binding free energy (Δ*G*_bind_) in three TCCs of similar length but different stability (PDB IDs: 2wpy, 4dzk, 1mof). Despite the nearly identical local coordination environment, the computed Δ*G*_bind_ values strongly depend on the overall protein structure, with variations in superhelical radius *R*_0_ upon ion removal systematically accompanying the observed binding thermodynamics. In particular, both the metastable TCC 2wpy—a variant of the GCN4 leucine-zipper domain previously shown to be unstable in the absence of chloride—and the synthetic design 4dzk exhibit highly unfavorable binding, suggesting that current biomolecular force fields may not fully capture either the stabilizing role of chloride or the conformational ensemble of the unbound state. By contrast, the calculated Δ*G*_bind_ in 1mof, a fragment of the MoMuLV retroviral transmembrane protein, is favorable and is associated with the presence of an additional C-terminal leash domain that modulates the binding-site environment. These results identify TCCs as critical benchmarks for improving the description of anion–protein interactions and the balance between bound and unbound states in future force-field developments.

## Graphical abstract

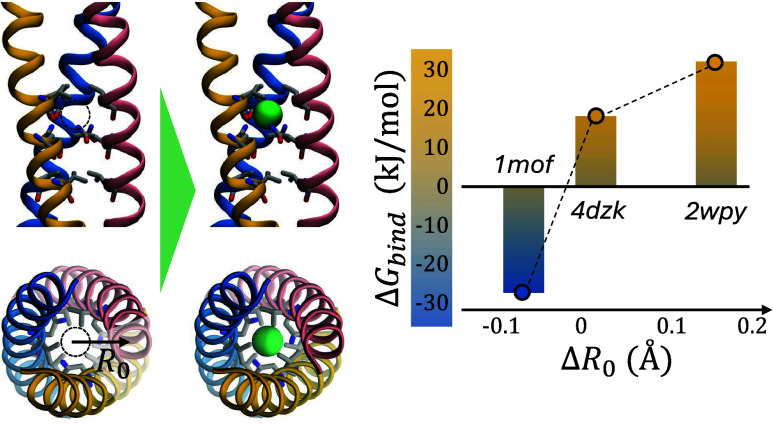

## Introduction

Coiled coils are superhelical structures formed when two or more α-helices wind around each other.^1–3^ They are ubiquitous structural motifs in proteins with roles in cellular signaling, gene regulation, and structural scaffolding. The superhelical arrangement commonly establishes a periodicity of 7 residues over two helical turns (i.e., amino acids n and n+7 occupy the same position in the plane perpendicular to the helical axis), though other periodicities are possible.^4,5^ In the most common case of the heptad repeat, the repeating positions of seven amino acids are denoted as (*a-b-c-d-e-f-g*). Positions *a* and *d*, at the interface between different helices, are commonly occupied by hydrophobic amino acids stabilizing the coiled-coil architecture.

However, polar and even charged amino acids can be found at *a* and *d*.^6,7^ Their presence presumably plays a role in protein dynamics and turnover in natural coiled coils, and in specifying a unique arrangement of the assembly, by destabilizing alternative combinations (for example, parallel trimers vs antiparallel dimers).^3^

In trimeric coiled coils (TCC), asparagine (N) or glutamine (Q) triads at position *d* or *a*, respectively, coordinating a buried chloride ion are a recurring feature.^8,9^ In addition to the amide-containing residues, the halide binding site in these systems is formed by a layer of branched hydrophobic amino acids like isoleucine (I) or valine (V)—at position *a* or *d* of the same heptad, for N and Q, respectively—resulting in the typical [I/V]­xxNxxx and Qxx­[I/V]­xxx heptads.

Starting from the GCN4 leucine zipper domain (GCN4-p1), Lupas and co-workers^10^ have engineered short (33 a.a.) sequences that form homotrimeric coiled coils with one, two, or three instances of Asn@*d*, each coordinating a chloride ion or other halides as revealed by their crystal structure. The insertion of each Asn@*d* layer was found to strongly reduce the stability of the trimers, and already with two Asn@*d* layers, the trimer did not form in solution but only under crystallization conditions. In addition, circular dichroism measurements at different ionic strengths of the peptides containing a single Asn@*d* layer revealed that the stability of the trimer depends on the presence of chloride ions.^10^ This ionic strength dependence is instead absent in the peptides, with a hydrophobic residue (Val) replacing the Asn@*d*.

Further instances of the salt-dependent structural stability of TCC with bound halides come from studies of viral fusion proteins. A 55-amino-acid fragment of MoMuLV retroviral transmembrane protein (TM) was structurally solved (pdb code: *1mof*) showing a central 33 a.a. coiled coil with a central chloride coordinated by the same Asn@*d* layer.^11^ In thermal denaturation experiments, the melting temperature of the trimer increased by ≃10 °C (from 64 to 75 °C) in the presence of Cl^–^ or Br^–^, but remained unchanged with F^–^. Furthermore, no halide effect was found in a variant with asparagine replaced by hydrophobic isoleucine. Similar observations were made for a soluble fragment of the transmembrane glycoprotein of the Ebola virus (pdb code: 2ebo).^12^

Salt concentration is known to play more general roles also in coiled coils without a central bound ion, by enhancing the hydrophobic effect at higher ionic strength and by screening electrostatic interactions such as intra- and interhelical salt bridges.^13–15^ However, in the Cl^–^-binding TCC described above (i.e., the GCN4-p1 variant and the coiled-coil fragments of MoMuLV and Ebola transmembrane glycoproteins) the reported salt-induced effects are absent in variants with the Asn@*d* replaced by a hydrophobic residue, pointing to a different mechanism, directly implicating the bound ion.

Being relatively simple and well-characterized structures, coiled coils serve as valuable model systems for both experimental and computational studies. Numerous molecular dynamics (MD) investigations have addressed their stability and dynamics, in an effort to understand the relationship between the interactions of amino acids at specific heptad positions—particularly the core ones—and the resulting stability of the oligomeric assembly (up to pentameric, but mainly dimeric and trimeric).^16–23^

The role of Asn@*a* and @*d* was addressed mainly in the context of dimeric CC,^24,25^ whereas dimeric and trimeric assemblies of the native GCN4-p1 peptide, containing a Asn@*a*, were studied by replica-exchange MD simulations with implicit solvent.^21^ In the GCN4-p1 trimer, the Asn@*a* layer coordinates an internal water molecule rather than an ion (pdb:4dme), and the side Asn side chain adopts a different conformation (rotamer) than the one in halide-coordinating TCC with Asn@*d*. An MD study of dimeric and trimeric CC under shear addressed the mechanical response of a TCC containing an internal Cl^–^-binding site of a Gln@*a* layer.^26^ To avoid potential artifacts due to the ion force field, however, the bound Cl^–^ was not included in the simulated system, and equilibrium MD simulations also showed a stable trimer in the absence of the ion.

Despite their frequent occurrence in both natural and designed structures, TCC domains with Cl^–^-coordinated Asn@*d* triads have not yet been explored by using computational molecular modeling. Here, we address this gap by performing extensive molecular dynamics simulations and free-energy calculations to investigate how binding of the central Cl^–^ influences trimer stability, and how the thermodynamics of Cl^–^ binding depend on the overall structure. Our investigation addresses the three TCCs reported in Figure 1: (i) the N16V/L19N GCN4-p1 variant by Lupas and co-workers,^10^ hereafter indicated by its PDB code *2wpy*, (ii) the fragment of the MoMuLV retroviral transmembrane protein (hereafter *1mof*)^11^ and (iii) CC-pII-I13N, a synthetic peptide designed by Woolfson and co-workers (hereafter *4dzk*).^9,27^ With respect to the shorter *2wpy* and *4dzk* sequences (33 and 32 a.a., respectively), *1mof* is longer (55 a.a.), though only 33 a.a. form the TCC domain (a.a. 46–78 in the sequence), whereas the C-terminal segment, called C-terminal “leash”, folds back into the grooves of the TCC core, in an antiparallel arrangement with respect to the α-helices. *2wpy* TCC is only marginally stable, with measured melting temperature (*T*_m_) of ≃ 30 °C at a peptide concentration of 100 μM, neutral pH, and [NaCl] = 25 mM. The other two TCCs are more stable (*T*_m_ > 50 °C). These three systems, differing in intrinsic stability though featuring a TCC portion of similar length, provide a coherent set to probe how the architecture of the coiled coil influences chloride binding and overall stability. Using MD simulations with enhanced-sampling and alchemical free-energy approaches, we investigate how the chloride-binding asparagine triad behaves dynamically and thermodynamically across these systems.

**Figure 1:**
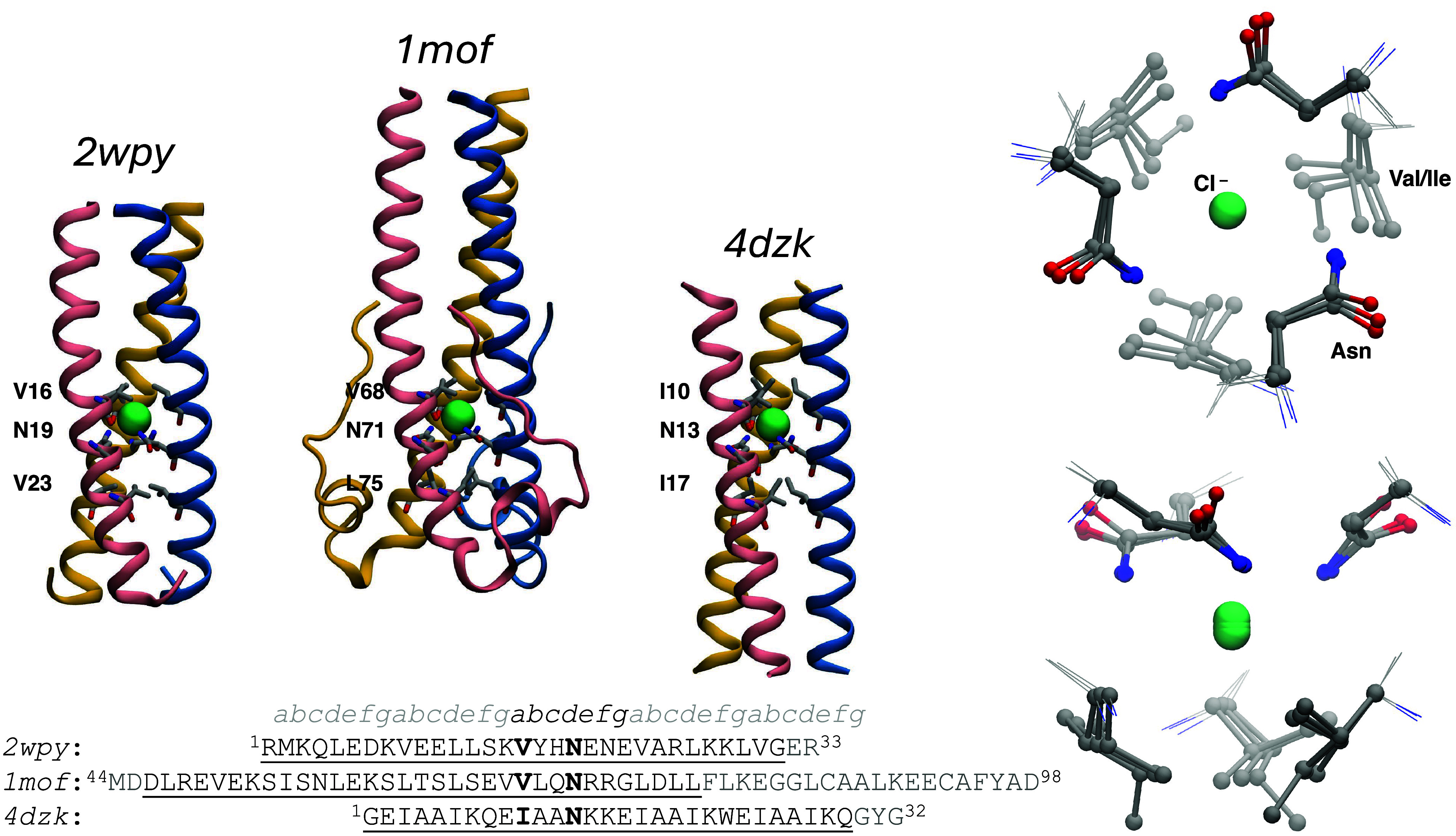
Structures of the TCC examined in this study. For each TCC the overall structure is shown in cartoon representation, and the amino acids in the layers adjacent to the Cl^–^-binding asparagine triad are shown in licorice. The three sequences are reported at the bottom and aligned with respect to the Cl^–^-binding asparagine. With respect to the wild-type MoMuLV transmembrane protein, the crystallized *1mof* sequence contains the C94A mutation, introduced to avoid potential intermolecular disulfide bond formation (the other two cysteines in the sequence being already involved in an intramolecular disulfide bond). The underlined amino acids are in coiled coil conformation, and the heptad position is indicated in the topmost line. The right panels show the superimposed Cl^–^-binding sites (bottom and lateral views).

## Methods

### Systems

The atomic coordinates for each of the three TCCs were obtained from the Protein Data Bank (PDB) using their respective entry codes. The missing C-terminal arginine was added to the X-ray structure of *2wpy*, and the peptides were capped with an acetyl group at the N-terminus to reproduce the experimental conditions.^10^ For *4dzk* we added the C-terminal Gly-Tyr-Gly that are missing in the X-ray structure and used standard NH_3_^+^ at the N-terminus and COO^–^ at the C-terminus. However, to rule out possible effects of terminal capping, we performed an alchemical run (see below) using the same capping as in ref 9, which has an acetyl group at the N-terminus and an NH_2_ group at the C-terminus. We denote this system as the *4dzk*-cap. Finally, for *1mof*, we directly used the X-ray structures with charged terminals, since no capping treatment is mentioned in the original experimental work.^11^ While *2wpy* is only marginally stable, and the model needed to match the exact construct used in experiments, *4dzk* and *1mof* are more stable and are not expected to be significantly affected by differences in terminal capping.

His18 in *2wpy* was protonated in the Nϵ, though also the doubly protonated state was examined (*2wpy*-H18^+^), while for all other amino acids in all three TCC, the standard protonation state at neutral pH was considered. The intrachain disulfide bridge between the two Cys in *1mof* (Cys86 and Cys93) was included in the model.

Crystallographic ions and water molecules were kept, and the TCC were solvated in a box of TIP3P water molecules, adding Na^+^ and Cl^–^ ions to neutralize the system and reach a concentration of ≃200–300 mM, as listed in Table 1. For TCC *2wpy* two settings were simulated to evaluate the effect of box geometry and size. The nominal peptide concentrations in the simulated systems exceed 4 mM, a much larger value than the experimental ranges (10–100 μM).^10,11,27^

**Table 1:** Settings for the Various Systems Examined^a^

name		peptide charge	box size (nm)	# of water molecules	# of Na/Cl
*2wpy*	cubic	0	10.2	35504	195/195
*2wpy*	tr. oct.	0	6.9	8010	44/44
*2wpy*-H18^+^	tr. oct.	+1	6.9	8010	41/44
*1mof*	tr. oct.	–3	8.8	17123	58/49
*4dzk*	tr. oct.	+1	7.2	9079	50/53
*4dzk*-cap	tr. oct.	+1	7.2	9080	48/51

a The box size in the case of the truncated octahedron geometry (tor octahedron) is the length of the vectors defining the box.

### MD Simulations

We used different force fields for simulating the molecular dynamics, CHARMM36m,^28^ Amber99sb-disp,^29,29,29^ and Amber14sb.^30^ With CHARMM36m, we used the pair-specific corrections (NBFIX) for the Lennard–Jones (LJ) interactions involving Cl^–^ and F^–^.^31^ Before production simulations, all systems were equilibrated starting with energy minimization (500 steps) and short MD simulations (1000 step with Δ*t* = 0.1 fs time step and 3000 steps with Δ*t* = 1 fs). The systems were then gradually heated to room temperature (298 K) with restraints on the heavy atoms (5000 kJ mol^–1^ nm^–2^), with five 200 ps-long runs at 100, 200, 250, and 300 K. The restraints were released with four 1 ns-long runs with decreasing force constants of 1000, 500, 100, and 50 kJ mol^–1^ nm^–2^. The equilibration procedure and subsequent runs used a Δ*t* = 2 fs and were performed in the NPT ensemble with the v-rescale thermostat^32^ for temperature control (coupling constant τ_*T*_ = 0.2 ps, with two separate thermostats controlling the protein and the rest of the system) and Parrinello-Rahman^33^ barostat for pressure control (coupling constant τ_*p*_ = 5 ps at 1 bar). Covalent bonds involving hydrogen atoms were constrained using the LINCS algorithm.^34^ Cutoff of 1.2 nm in the case of CHARMM36m and 1.0 nm for Amber14sb and Amber99sb-disp were used for nonbonded interactions, and long-range electrostatic interactions were treated with Particle Mesh Ewald.^35^ Some of the runs (normal MD and metadynamics, but not alchemical simulations) were performed using the method of hydrogen mass repartitioning (HMR), allowing for a longer time step (4 fs).^36^ With HMR, all covalent bonds were constrained with LINCS. All simulations were performed using GROMACS (v. 2022 and 2024).^37^

### Replica Exchange Metadynamics

Replica exchange 2D metadynamics simulations were run on the “*2wpy* tr. oct.” system (see Table 1) for 8 *μs* with 40 replicas spanning a 298–397 K temperature range. HMR was used (see above) and exchange between neighboring replica was attempted every 500 MD steps (2 ps).

2D metadynamics was implemented using the open-source, community-developed PLUMED library^38^ (version 2.8). The first collective variable (CV) is the coordination between any chloride ion and any of the three Asn@*d* (one for each of the three monomers) of the binding site. The PLUMED COORDINATION CV was used, defined as

1 $$c=\sum_{i\in A}\sum_{j\in B}\frac{1-{\left(\frac{r_{\mathrm{ij}}-d_{0}}{r_{0}}\right)}^{6}}{1-{\left(\frac{r_{\mathrm{ij}}-d_{0}}{r_{0}}\right)}^{12}}$$

where set *A* contains all the Cl^–^ ions in the system and set *B* contains the ND2 atom of each of the three Asn@*d*, *r*_0_ = 0.5 nm and *d*_0_ = 0.1 nm using a neighboring list with a cutoff of 2 nm updated every 100 steps. When the coordination is 3, the Cl^–^ is bound to the coiled coil, and when it is 0, the unbound state is sampled. We verified that the contribution of states in which three different Cl^–^ ions are coordinated to the three Asn is negligible, i.e., the states with coordination ≃3 almost always correspond to a single Cl^–^ in a well-folded binding site. The second CV, interface Cα dRMSD, monitors the stability of the coiled coil by calculating the deviation (root-mean-square distance) of intermonomer Cα distances from the X-ray structure. The PLUMED DRMSD CV was used, including all intermonomer distances (TYPE = INTER-DRMSD) with the Cα of residues at the interface (position *a* and *d*, from position 5 to 30 in the sequence) within an upper cutoff of 7 nm. To avoid complete denaturation of the trimer, a fourth-order restraint was put on this CV (UPPER WALLS) at values larger than 0.5 nm, with a force constant (KAPPA) of 5000 kJ mol^–1^ nm^–4^. The well-tempered variant of metadynamics was used,^39^ with a biasfactor of 15. The initial height of the 2D Gaussian hill was set at 0.014285 *k*_B_T, according to each replica’s temperature, with a sigma of 0.02 (unit-less) for the Cl^–^ coordination CV and 0.005 nm for the interface Cα DRMSD. Each replica was simulated for 8 μs.

### Binding Free-Energy Alchemical Calculations

For the calculation of Cl^–^-binding energies, we used the double-decoupling method (DDM).^40,41^ As depicted in Figure 2 and with reference to our system, the method consists of switching off the interactions of Cl^–^ with its environment in two distinct contexts: the ion in the solvent, resulting in (the opposite of) the ion hydration free energy (Δ*G*_Cl →0,sol_ = −Δ*G*_hyd_), and the ion within the protein binding pocket (Δ*G*_Cl→0,prot_). The system in the first case is a box of solvent with the (disappearing) ion, whereas in the second case, the properly solvated protein and the ion in its binding pocket are both present. The binding free energy is then given by

2 $$\Delta G_{\mathrm{bind}}=\Delta G_{\mathrm{Cl}\to 0,\mathrm{sol}}-\Delta G_{\mathrm{Cl}\to 0,\mathrm{prot}}$$

**Figure 2:**
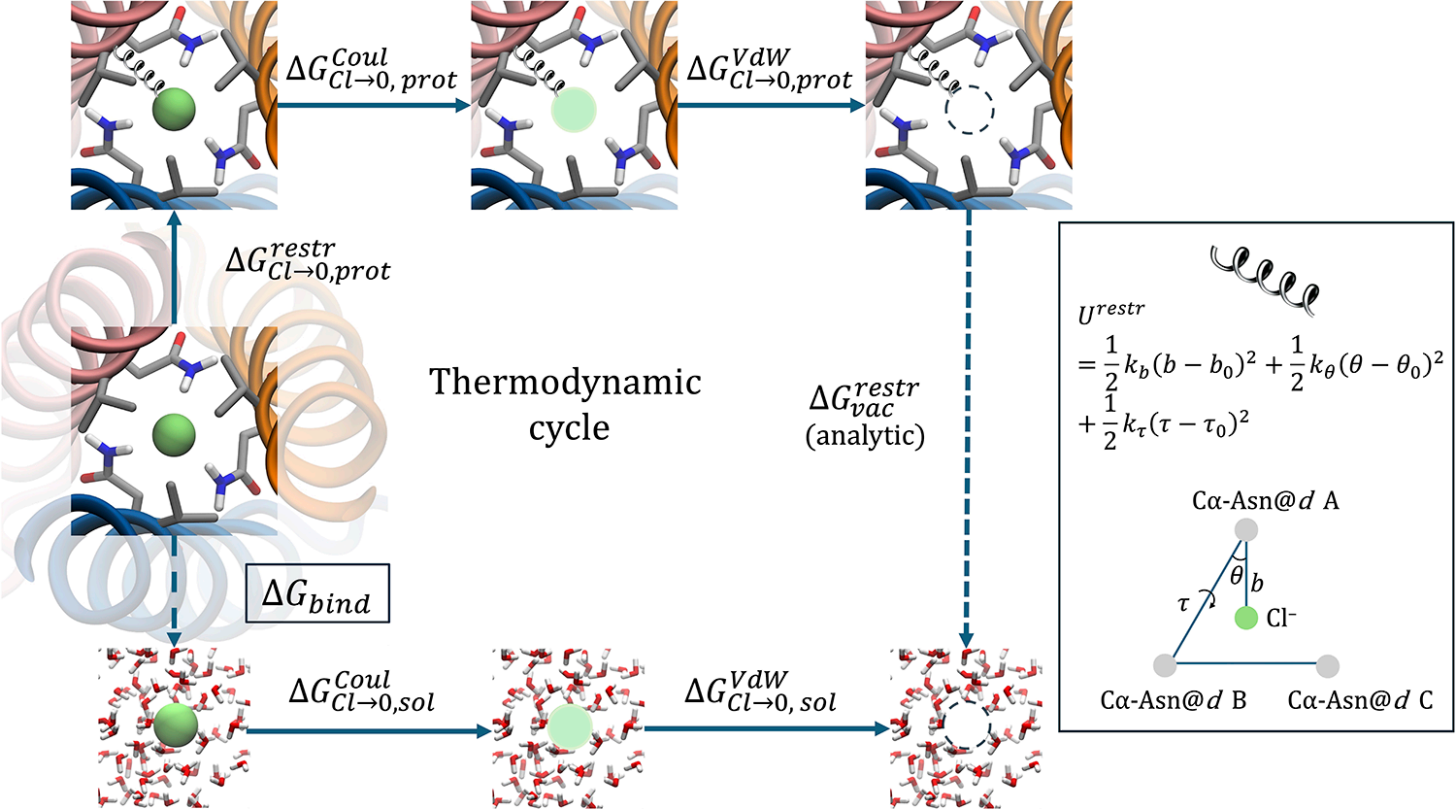
Thermodynamic cycle associated with the double-decoupling method. The left panel shows the restraint applied to the particle in order to avoid migration outside the binding site (eq 3).

The two processes were carried out using alchemical free-energy methods, in which the interactions of the ion with its environment are gradually “turned off” through a coupling parameter in the Hamiltonian, i.e., *H* = (1−λ)*H*_full_ + *λH*_decoupled_. The decoupling is performed in various stages, with electrostatic interactions being decoupled first, followed by van der Waals terms (dispersion and repulsion, represented in the usual Lennard-Jones form). For the vdW decoupling stage, we used the soft-core potential with α = 0.5.^42^ The free energy associated with each stage is then calculated using thermodynamic integration (TI), i.e.,

$$\Delta G=\int_{0}^{1}\left\langle \frac{\partial H}{\partial \lambda }\right\rangle d\lambda$$

We used the traditional trapezoidal rule to evaluate this integral and verified that, within the statistical uncertainties, the results from TI are consistent with those obtained using the multistate Bennett acceptance ratio method (BAR),^43^ as implemented in the GROMACS *bar* module.

The process for Δ*G*_Cl →0,prot_ requires an additional step, because at the end of the alchemical transformation the dummy particle is free to wander in a highly inhomogeneous system (this is not the case for the first process, Cl→0, sol, as the solvent is uniform and isotropic). To avoid sampling issues associated with this wandering, restraints are turned on so that the dummy particle is confined within the protein binding pocket.^40,41^ The free energy Δ*G*_restr_ associated with turning off the restraints on the dummy particle is calculated analytically (see below).

In the Δ*G*_Cl→0,sol_ calculation, the electrostatic interactions were first turned off using 14 windows with λ_Coul_ = 0.0, 0.01, 0.05, 0.11, 0.21, 0.32, 0.44, 0.56, 0.68, 0.79, 0.89, 0.95, 0.99, 1.0, and then the vdW interactions were turned off using the same 14 λs windows. We first equilibrated each window with 1 ns MD serially (i.e., each replica bar the first one started from the end of the previous alchemical step) and then performed 5 ns production runs. The results for this transformation converged very quickly.

To calculate Δ*G*_Cl →0,prot_ we first turned on the restraints (defined below) using 8 windows with λ_restr_ = 0.0, 0.1, 0.2, 0.3, 0.5, 0.7, 0.9, 1.0, then turned off the electrostatics using 18 windows with λ_Coul_ = 0.0, 0.05, 0.1, 0.15, 0.2, 0.26, 0.32, 0.39, 0.46, 0.54, 0.61, 0.68, 0.74, 0.8, 0.85, 0.9, 0.95, 1.0, and finally turned off the vdW using 16 windows with λ_vdW_ = 0.0, 0.1, 0.2, 0.29, 0.38, 0.47, 0.55, 0.63, 0.7, 0.77, 0.83, 0.89, 0.93, 0.97, 0.99, 1.0. The λ values listed represent the final scheme adopted and are a practical compromise between accuracy and efficiency. In earlier runs, slightly different λ sets were employed, but these variations did not affect the resulting free-energy values.

Each of the replicas were equilibrated for 1 ns serially, and the first replica started from the final snapshot of plain MD simulations. Production runs lasted 50 ns and were done with Replica Exchange.^44^ Exchanges between adjacent replicas were attempted in each 500 steps, alternating the pairs being considered for exchange. The combination of free-energy perturbation with replica exchange leads to better sampling and improved convergence of the calculations.^45^ Uncertainties on the resulting values were estimated using a block averaging (binning) procedure: for each production run, the first 10 ns were discarded as additional equilibration, and the remaining 40 ns were divided into five blocks. The standard deviation across the block averages was taken as the statistical error. When more than one production run was performed on the same system (either by starting with a different set of random velocities or by using the configurations of a previous run as starting points), all block averages were grouped in one set for statistical analysis. The details of each run are reported in the Supporting Information (Table S4–S6).

The restraints on the Cl^–^/dummy particle are shown in Figure 2 and are defined as a combination of harmonic potential on the distance *b* between the bound Cl^–^ and Cα of the Asn@*d* of chain A (Cα-Asn@*d*A); on the angle θ between Cα-Asn@*d*B, Cα-Asn@*d*A and Cl^–^; and on the dihedral torsion angle τ defined by Cα-Asn@*d*C, Cα-Asn@*d*B, Cα-Asn@*d*A and Cl^–^. The associated potential energy is thus

3 $$U^{\mathrm{restr}}=\frac{1}{2}k_{b}{\left(b-b_{0}\right)}^{2}+\frac{1}{2}k_{\theta }{\left(\theta -\theta_{0}\right)}^{2}+\frac{1}{2}k_{\tau }{\left(\tau -\tau_{0}\right)}^{2}$$

with *k*_*b*_ = 5000 kJ/nm^2^, *k*_θ_ = 500 kJ/rad^2^, *k*_τ_ = 500 kJ/rad^2^, and *b*_0_ = 0.52 nm, θ_0_ = 40.2°, τ_0_ = −44.6° for *2wpy* and *1mof*. For *4dzk* we used θ_0_ = 37.5° and τ_0_ = −48.0°, while all of the other parameters were the same. The reference values are the averages of the corresponding quantities during plain MD simulations, and the force constants are selected to mimic the width of the distributions. Our choice of the Cα atoms instead of, for instance, the closer amino group nitrogen atoms was dictated by the high mobility of the Asn side chains in the decoupled states in particular.

Δ*G*_vac_^restr^ is calculated as^41,46^

4 $$\Delta G_{\mathrm{vac}}^{\mathrm{restr}}=-\mathrm{RT}\ln\frac{V_{0}\sqrt{k_{b}k_{\theta }k_{\tau }}}{{\left(2\pi k_{B}T\right)}^{3/2}b_{0}^{2}\sin\theta_{0}}$$

where *V*_0_ = 1.661 nm^3^ is the standard volume for a one molar standard state, *R* is the gas constant.

The complete expression for the free energy associated with the decoupling of the ion in the protein is given by

5 $$\Delta G_{\mathrm{Cl}\to 0,\mathrm{prot}}=\Delta G_{\mathrm{Cl}\to 0,\mathrm{prot}}^{\mathrm{Coul}}+\Delta G_{\mathrm{Cl}\to 0,\mathrm{prot}}^{\mathrm{vdW}}+\Delta G_{\mathrm{Cl}\to 0,\mathrm{prot}}^{\mathrm{restr}}-\Delta G_{\mathrm{vac}}^{\mathrm{restr}}$$

In selected cases, indicated with 0 →Cl, we performed the additional backward transformation to address potential hysteresis issues, i.e. starting from the fully decoupled state (from the plain simulation with unbound Cl^–^) with restraints and gradually switching on first the vdW and then the electrostatic interactions and finally switching off the restraints, using the same λ values but in the reverse order.

In the case of *1mof*, we performed two additional transformations: Cl→F and Cl→W, i.e., the bound Cl^–^ is transformed into, respectively, a fluoride ion F^–^ and a water molecule, both in the protein and in a solvent box. Since the F^–^ and the water molecule remain confined within the protein Cl^–^-binding site, the restraints are not necessary. For the Cl → F transformation the only term needed is the vdW term Δ*G*_Cl→F,env_^vdW^—env being either the protein (prot) or a solvent box (sol)—and was calculated using λ_vdW_ = 0.0, 0.01, 0.05, 0.10, 0.16, 0.23, 0.32, 0.43, 0.57, 0.68, 0.77, 0.84, 0.90, 0.95, 0.99, 1.0. This transformation converges very quickly also in the protein, and 5 ns simulations were sufficient.

For the Cl→W transformation both Δ*G*_Cl→W,env_^Coul^ and Δ*G*_Cl→W,env_^vdW^ are needed. In this case we defined the starting state as the Cl^–^ with two bound dummy atoms and the end point as a TIP3P molecule, and first transformed the LJ interactions of the Cl^–^ to those of TIP3P oxygen (the LJ parameters of TIP3P hydrogen are zero) using λ_vdW_ = 0.0, 0.12, 0.24, 0.36, 0.48, 0.6, 0.72, 0.8, 0.85 0.9, 0.95, 1.0 and then the electrostatic part by using λ_Coul_ = 0.00, 0.09, 0.18, 0.27, 0.36, 0.45, 0.54, 0.63, 0.72, 0.80, 0.88, 0.95, 1.00. In this case, the binding free energy referred to the Cl^–^ standard state is given by^47,48^

6 $$\Delta G_{\mathrm{bind}}=\Delta G_{\mathrm{Cl}\to W,\mathrm{sol}}-\Delta G_{\mathrm{Cl}\to W,\mathrm{prot}}+\mathrm{RT}\ln\left[H_{2}O\right]/c_{0}$$

where the last term in the RHS takes into account the concentration of bulk water and is simply given by *RT* ln 55.5 = 9.95 kJ/mol.

### Coiled-Coil Crick Parameter Fitting

In both plain MD simulations and some alchemical runs, we monitored selected coiled-coil Crick parameters^49^ computed from MD-averaged structures. The parameters were obtained using the CCCP tool developed by Grigoryan and DeGrado,^50^ which extracts Crick parameters from atomic coordinates by fitting an idealized coiled-coil geometry to the helical backbone. The parameters are varied until a minimum RMSD between the idealized coiled coil and the input structure is found. Fits were performed using either the full TCC backbone coordinates or only the heptad containing the Asn@*d* residue; in the latter case, we refer to the resulting quantities as *local* parameters. A schematic depiction of the Crick parameters is provided in Figure S8 and is described in the corresponding caption. In the case of plain MD simulations, uncertainty estimates were obtained by dividing each trajectory (excluding the first 100 ns) into three contiguous segments, computing the coiled-coil parameters from the average structure of each 300 ns segment, and reporting the standard deviation across the resulting set.

## Results

### Flexibility and Binding Site Geometry during MD Simulations

We performed plain 1 μs simulations using CHARMM36m with the NBFIX correction of *2wpy*, *4dzk*, and *1mof* (tr. oct. box in Table 1) to monitor the stability and dynamics of the structures and the geometry of the Cl^–^-binding site. For each system, we run one MD trajectory starting with the internal Cl^–^ as in the X-ray structures and the other with the Cl^–^ replaced by a water molecule. In the case of *1mof* we run additional simulations to better characterize the leash mobility, which are discussed in the following. No Cl^–^ binding or unbinding event was detected during the simulated time, while the internal water molecule is mobile in *2wpy* and *4dzk* but not in *1mof* (see below). The simulations were also performed for *2wpy*-H18^+^ and for *2wpy* with a cubic box. The results regarding the RMSD and RMSF analyses are completely analogous to the case of *2wpy* tr oct., so they are not discussed here.

The results are reported in Figure 3 (see also the Supporting Information, Figures S1 and S2). For all systems, the fluctuations of the TCC domain show only moderate differences between the Cl^–^ bound and unbound states. As expected, the central region is more rigid than the terminals. In particular, the C-terminal leash region in *1mof* (a.a. 79–98) is more mobile, and in the Cl^–^-unbound trajectory we observed a transient unfolding event involving this region, resulting in high fluctuations of the leash for one of the monomers (Figure 3, top-left panel). As reported in the next subsection, these higher fluctuations are recurring features in both the Cl^–^ bound and unbound states.

**Figure 3:**
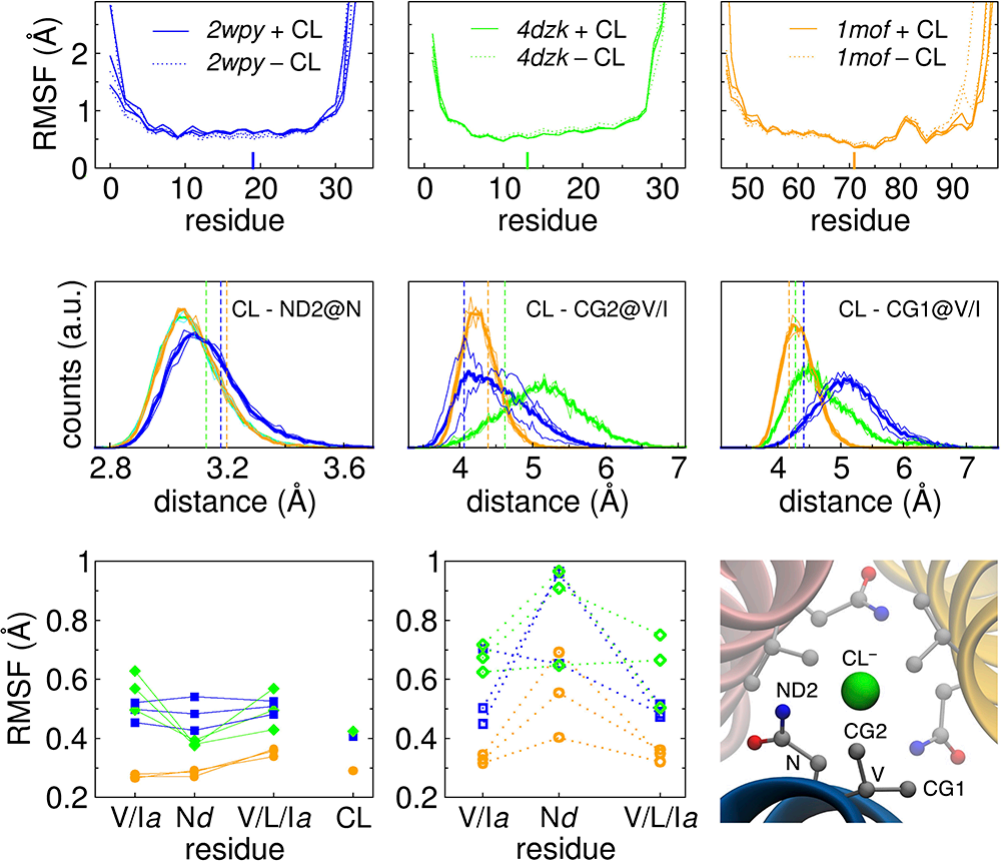
Top panels: Per-residue backbone root-mean-square fluctuations (RMSF) with respect to the average structure for each of the three analyzed TCC with (solid lines) and without (dotted lines) bound Cl^–^. Each line corresponds to one monomer. The short bar indicates the position of the Asn@*d*. The analysis was performed on the second half of the trajectory (0.5–1 μs). Center panels: Distributions of distances of selected atoms from the central Cl^–^ (same color code as top panels). The thin lines are the distributions for each monomer in the trimer, while the thick lines are the average. The dashed vertical lines are the corresponding values from the X-ray structures. The binding site is shown in the right bottom panel, with labels on the atoms involved in the plotted distance distributions. *4dzk* contains a Ile instead of Val in the *a* layer preceding the Asn@*d*. The CD of Ile was not considered in the analysis because it points away from the Cl^–^. Bottom panels: RMSF analysis restricted to the Asn@*d* and adjacent *a* layers (V16, N19, and V23 for *2wpy*, I10, N13, and I17 for *4dzk*, and V68, N71, and L75 for *1mof*), with (left panel) or without (center panel) the Cl^–^ ion.

Overall, the core of the TCC is more rigid in the case of *1mof* than in the other two systems, and the fluctuations of the backbone in the Cl^–^-binding portion are around 0.35 Å; compared with 0.5–0.6 Å; of *2wpy* and *4dzk*. While in the cases of *1mof* and *4dzk* the fluctuations in this region are slightly lower in the Cl^–^-bound case than in the Cl^–^-unbound cases, the opposite is true for *2wpy*.

The distributions of distances between the central Cl^–^ ion and key atoms in the binding pocket during the MD simulations are shown in the middle panels of Figure 3 and reveal that the Asn amide nitrogen is slightly closer in *1mof* and *4dzk* (the distribution in these two cases is indistinguishable) than in *2wpy*. The same is true for the γ carbon atoms of the Val residue in direct contact with the Cl^–^ (*4dzk* contains an Ile in that position, so the comparison is not possible), which also show a much broader distribution in *2wpy*.

We also monitored the flexibility of the Cl^–^ binding site, by calculating the fluctuations restricted to the Asn@*d* layer, the two adjacent *a* layers, and the Cl^–^ ion when present. Again, the case of *1mof* stands out for its higher rigidity, particularly in the Cl^–^-bound case. This higher rigidity is a consequence of the additional C-terminal leash, which is involved in hydrophobic, polar, and salt-bridge interaction with the TCC core, as discussed in detail in the following.

Additional simulations of *2wpy* with Amber99sb-disp and CHARMM22* were performed on longer timescales (see Supporting Information, Figure S3). Also, with these two force fields, which give a more balanced description of folded vs disordered proteins, no sign of structural destabilization in the absence of the bound Cl^–^ could be detected (up to 10 μs in the case of CHARMM22*), and no Cl^–^ binding took place. We found, however, that with CHARMM22* the Cl^–^ is less stably bound to the TCC, and can easily exit the binding pocket.

### Water Molecules in the Binding Site

The internal regions of *2wpy* and *4dzk* are permeable to water molecules, and rather frequent exchanges with the bulk are observed, while *1mof* shows much slower water exchange on the simulated time scale. In the Cl^–^-unbound state, the internal water exchanges within the first 6 ns in *2wpy* and 50 ns in *4dzk*, while no exchange was detected for *1mof* for the whole 1 μs trajectory. Thereby, for this system, we performed a simulation starting with an empty Cl^–^-binding pocket and found that it took 450 ns for a water molecule to enter.

Thanks to the flexible nature of the Asn side chain, and the ability of the amide group to act both as a donor and an acceptor of hydrogen bonds, when the Cl^–^ is absent interactions are established either between the Asn and the water molecule, or among the Asn themselves when the water molecule is absent (Figure S1).

Figure 4 reports the isosurfaces of water density and the corresponding water-occupancy statistics for *2wpy* and *4dzk* in both the Cl^–^-bound and unbound states. Water molecules enter the binding site even when Cl^–^ is present, where they localize in the region between the Asn@*d* layer and the following Val or Ile residue at position *a*. In the Cl^–^-unbound state, the binding site of *2wpy* remains mostly empty, whereas it is more frequently occupied by water in *4dzk*. Conversely, when Cl^–^ is bound, water molecules enter the binding site more often in *2wpy* than in *4dzk*.

**Figure 4:**
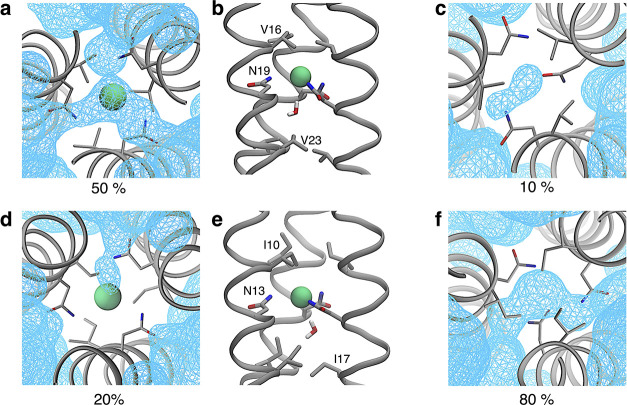
(a) Binding site of *2wpy* in the Cl^–^-bound state, showing the water number density sampled during the MD simulation. The isosurface (light-blue wireframe) is shown at a density value of 0.1 and was computed over the 0.5–1 μs time window using the VolMap tool of VMD.^51^ The percentages reported at the bottom of this and all other panels indicate the water occupancy of the site, defined as the fraction of trajectory frames containing one or more water molecules (first significant digit only). (b) Representative snapshot corresponding to panel a, highlighting the position of the additional water molecule. (c) Same as panel (a), but for the Cl^–^-unbound state. (d–f) Same as panels a–c, respectively, for *4dzk*.

Given the slow water exchange in *1mof* we also addressed the case of an additional water molecule in the Cl^–^-bound state. While this molecule remains stably bound inside *1mof* for the whole 1 μs simulation, its binding is thermodynamically unfavorable as revealed by its highly positive Δ*G*_bind_ calculated by alchemical transformation below.

### Leash Mobility in *1mof*

The presence of the leash adds complexity to the structure of *1mof*. Here, we investigate in more detail its mobility during additional 1 μs simulations of both Cl^–^-bound and -unbound states. In total, ten MD simulations were performed, five with bound chloride (MD0–4_+Cl_) and five without chloride (MD0–4_–Cl_), starting from the equilibrated structure with different sets of random velocities. The MD0 trajectories correspond to the simulations discussed above.

We distinguish two subregions within the leash (Figure 5). The first corresponds to the C-terminal portion (residues 94–98; hereafter *leash-ter*), which interacts rather weakly with the coiled-coil portion. The second subregion (residues 80–93; hereafter *hinge*) interacts more strongly and contains an intrachain (and intraleash) disulfide bond between Cys86 and Cys93 and a short α-helical segment. The hinge has the structural role of reversing the direction of the polypeptide chain. The whole leash interacts with residues both on the same polypeptide chain (*i*) and on the next chain (*i* + 1) along the superhelix (e.g., the leash of chain A contacts the coiled-coil region of chain B, and analogously for B→C and C→A).

**Figure 5:**
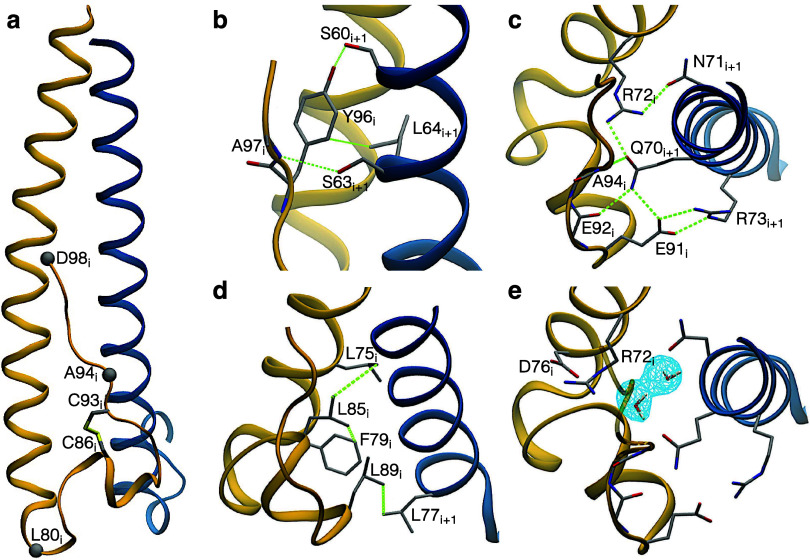
(a) Leash of *1mof* and its positioning at the interface of helix *i* in yellow and *i+1* in blue. The gray spheres correspond to the α carbon atoms of residues used to define the leash subregion, the hinge, from residue 80 to 93, and the leash-ter, from 94 to 98. The two cysteine residues involved in the disulfide bridge are explicitly shown. This and panels b, c and d are from the X-ray structure (PDB:1mof). (b) Interactions of the leash-ter region with α-helix *i*+1. (c) Hydrogen bonds and salt bridges between the hinge region (residue Glu91_*i*_, Glu92_*i*_, Ala94_*i*_ are shown) and the coiled-coil core (Arg72_*i*_, Gln70_*i*+1_, Asn71_*i*+1_, Arg73_*i*+1_). (d) Hydrophobic cluster involving the hinge region (Leu85_*i*_ and Leu89_*i*_) and the coiled-coil core (Leu75_*i*_, Phe79_*i*_ and Leu77_*i*+1_). (e) Representative snapshot from an MD simulation with bound Cl^–^, showing the repositioning of Arg72_*i*_, and its contact with Asp76_*i*_. The two water molecules replacing the arginine side chain are shown, together with the isosurface (light-blue wireframe) of the water number density at a value of 0.5, calculated over the trajectory using the VolMap plugin of VMD.^51^

In the X-ray structure, the interface between leash-ter and the proper TCC portion features interactions between Ala97_*i*_ carbonyl backbone and Ser63_*i*+1_ side chain, and between Tyr96_*i*_ and Ser60_*i*+1_, via its phenolic group, and Leu64_*i*+1_, through a hydrophobic contact with its aromatic ring (Figure 5b). Furthermore, a central role is played by Gln70_*i*+1_, located on the TCC at position *c*, just before Asn@*d* (Figure 5c). The amide group of Gln70_*i*+1_ is at a hydrogen-bond distance with the leash backbone (carbonyl oxygen of Glu92_*i*_ and amide nitrogen of Ala94_*i*_). Gln70_*i*+1_ also interacts with Arg72_*i*_, which is in turn hydrogen-bonded to the side-chain amide oxygen of Asn71_*i*+1_ (the Asn@*d*). Additional stabilizing contacts include hydrophobic interactions between Leu85_*i*_ and Leu89_*i*_ of the hinge and Leu75_*i*_ (@*a*), Leu77_*i*_ and Phe79_*i*_ of the coiled-coil core (Figure 5d).

Panel (e) of Figure 5 shows a configuration that is most frequently sampled across the MD trajectories. Arg72_*i*_ predominantly loses contact with Asn71_*i*+1_ in both the Cl^–^-bound and unbound states, although transient reformation of this interaction is observed. Instead, Arg72_*i*_ is most often in contact with Asp76_*i*_, while the space left empty by its displacement is occupied by water molecules. Depending on the leash conformation, these waters are either trapped between the leash and the TCC core when the leash is closed or located at the interface between the protein and the bulk solvent when the leash is open.

We monitored the stability of these interactions during the MD simulations by calculating the minimum distances between the corresponding residue pairs. The distributions (reported in Figure S4) differ between the Cl^–^-bound and unbound states, mostly for the interactions involving Arg72. This is not surprising because when the central Cl^–^ is absent, two of the three Asn71 side chains flip, presenting the NH_2_ group instead of the carbonyl to the outside of the coiled coil, thereby interfering with the hydrogen bond network between Arg72_*i*_, Gln70_*i*+1_, and nearby water molecules.

To monitor the fluctuation of the leash, we calculated the dRMSD from the X-ray structure of sets of distances involved in the interface between the leash and the coiled-coil core, for both leash-ter and hinge (for the full traces see Figures S5 and S6). The histograms shown in Figure 6 were computed by pooling all MD trajectories (considering only the second half of each simulation). Leash fluctuations are observed in both the Cl^–^-bound and unbound states and frequently lead to partial detachment of the terminal leash segment (leash-ter) from the coiled-coil core, as indicated by the subpopulation with dRMSD values in the 0.7–1.1 nm range. By contrast, the hinge region is generally more stable, with dRMSD values below 0.5 nm in the vast majority of counts. In one of the Cl^–^-unbound trajectories (MD2_–Cl_), the hinge region also loses contacts with the coiled-coil core, resulting in a more extensive detachment of the leash and giving rise to the high-dRMSD subpopulation highlighted in the insets. We will further characterize this open-leash configuration in terms of its Cl^–^ binding free energy.

**Figure 6:**
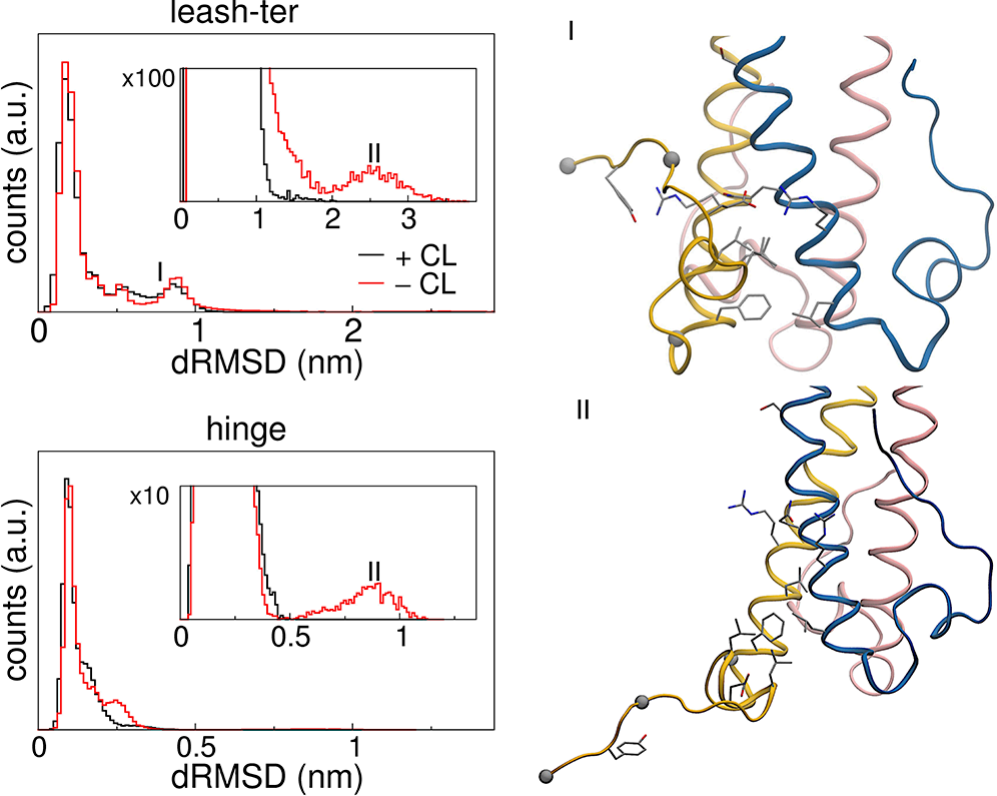
Right: Histograms of dRMSD of the interface between the coiled-coil core and the leash-ter region (residues 94_*i*_–98_*i*_) or the hinge region (residues 80_*i*_–93_*i*_). The dRMSD was computed over all non-hydrogen atom pairs that are within 0.5 nm in the X-ray structure, with one atom belonging to the *leash*-ter region and the other to the interface residues of the coiled-coil core (72_*i*_, 75_*i*_, 76_*i*_, 79_*i*_, 70_*i*+1_, 71_*i*+1_, 73_*i*+1_, and 77_*i*+1_). The histograms were computed by pooling all MD trajectories and considering only the second half of each simulation. Left: representative structure with open leash-ter (I) and with a completely open leash (II).

Regarding fluctuations in the binding-site region, the plots corresponding to those shown in Figure 3 are reported separately for each simulation in the Supporting Information (Figure S7). We note that simulations MD1–4_+Cl_—MD4_+Cl_ in particular—exhibit larger RMSF values for Leu75 compared with MD0_+Cl_. This behavior correlates with an increased instability of the hinge contacts (Figure S5). Consistently, in the Cl^–^-unbound simulation MD2_–Cl_, the RMSF of Leu75 on the open-leash chain reaches the highest value observed across all simulations.

Overall, a statistically meaningful characterization of these leash rearrangements would require either substantially longer simulation timescales or targeted enhanced-sampling approaches. In the MD simulations presented here, we do not observe a robust correlation between Cl^–^ binding and leash fluctuations; however, this possibility remains open and requires further investigation through dedicated computational and experimental studies.

### Local Coiled-Coil Crick Parameters

We performed an analysis of the generalized Crick helical parameters^49,50^ of the MD averaged structures (main chain only) in the Cl^–^-bound and unbound states of the three TCCs, using the cccp structure fitter available online.^52^ The full results are reported in Table S1. Here we report in Table 2 the values of the superhelical radius *R*_0_—defined as the distance from the superhelical axis to the axis of each α-helix—extracted for the heptad containing the Asn@*d*. This local *R*_0_, which measures the tightness of the trimer at the Cl^–^-binding site, shows subtle but relevant variations among the three systems and between the Cl^–^-bound and -unbound states of the same TCC.

**Table 2:** Local Superhelical Radius *R*_0_ (in Å) in the MD Average Structures^a^

system	*R*_0_ + CL^b^	*R*_0_–CL	Δ*R*_0_
*2wpy*	6.24 (0.01)	6.07 (0.02)	0.17 (0.02)
*2wpy* cubic	6.24 (0.02)	6.08 (0.03)	0.16 (0.04)
*2wpy*-H18^+^	6.18 (0.01)	6.05 (0.01)	0.13 (0.02)
*4dzk*	6.32 (0.01)	6.30 (0.01)	0.02 (0.02)
*1mof* ^c^	6.03 (0.03)	6.10 (0.02)	–0.07 (0.04)
*1mof* open-leash	–	6.04 (0.01)	–

a The heptad containing the Asn@*d* is considered (*2wpy*: residues 16–22; *4dzk*: residues 10–16; *1mof*: residues 68–74).

b Values in parentheses are uncertainties obtained as described in the Methods section.

c For each state, the replica simulations are grouped together, apart from the one with an open leash (MD2_–CL_), which is reported in the next row.

The bound state of *1mof* has the shortest *R*_0_ signaling that the Asn@*d*-containing heptad is more compact in *1mof* than in the other two TCCs. The higher values of *4dzk* in both states are presumably a consequence of the presence of isoleucine at position *a* before Asn@*d*, which is bulkier than Val@*a* present in *2wpy* and *1mof*.

The *R*_0_ values indicate a tightening of the TCC upon Cl^–^ removal in the case of *2wpy*, consistently with both the prevalent presence of an additional water in the binding site in the Cl^–^-bound state, and low water occupancy of the binding site in the Cl^–^-unbound state. The simulations of *2wpy* with the other force fields confirm the trend (Table S2): *R*_0_ decreases from 6.42 Å in the bound state to 6.14 Å in the unbound state using Amber99sb-disp and from 6.24 to 6.07 Å using CHARMM22*.

By contrast, *1mof* exhibits an increase in *R*_0_ upon ion removal. While we were unable to identify the molecular determinants of this behavior, a comparison across individual *1mof* trajectories suggests a dependence on the leash conformation. In particular, when the leash adopts an open configuration, the unbound-state *R*_0_ tends to decrease (see Table S3 for per-trajectory values). In the simulation MD2_–Cl_, where the leash becomes fully detached, *R*_0_ reaches the lowest value observed for the unbound state (6.02 Å). However, *R*_0_ is already relatively low at the beginning of this trajectory (6.04 Å) when the leashes are still associated with the TCC, arguing against a simple causal link between leash detachment and superhelical tightening. Given the anomalous behavior of MD2_–Cl_, also in terms of Cl^–^ binding thermodynamics (see below), this trajectory was excluded from the averages reported in Table 2. The simulations of the Cl^–^-bound state also show some variability of the *R*_0_ parameter, with trajectory MD2_+Cl_ reaching the highest value (6.09 Å) in the middle segment of the simulation. The hinge region correspondingly shows a high dRMSD (Figure S5). By contrast, in simulation MD0_+Cl_, where the dRMSD shows a more stable hinge region, the *R*_0_ is the shortest and also shows minimal variations across the time segments.

### Energetics of the GCN4-Derived Coiled Coil (*2wpy*) from Replica-Exchange Metadynamics

We investigated in more detail the free-energy landscape of *2wpy*, using two-dimensional metadynamics with replica exchange. The two collective variables (CVs) were chosen to address the interplay between chloride binding, using the coordination number of the Cl^–^ to the three Asn19 (see eq 1), and stability of the coiled coil, using the RMSD of the central interface Cα. During the trajectory (8 μs × 40 replicas), several events of chloride binding and unbinding were detected, in contrast with the plain MD case in which no such event took place.

Figure 7 shows the 2D potential of mean force (PMF), together with the integrated 1D PMF for Cl^–^ coordination at different temperatures. Surprisingly, the Cl^–^-bound state is *higher* in energy than the unbound state by several *k*_B_T units. The possibility of detecting Cl^–^ binding-related stabilization is thus unfeasible, as the lowest free-energy state is not the folded structure with the bound ion. The energy difference between the bound and unbound state (i.e., Δ*G*_bind_) results ≃38 kJ/mol at 298 K. Considering the Cl^–^ concentration in these simulations (≃200 mM), the predicted Δ*G*_bind_ value for the standard state ([Cl^–^] = 1 M) would be 34 kJ/mol.

**Figure 7:**
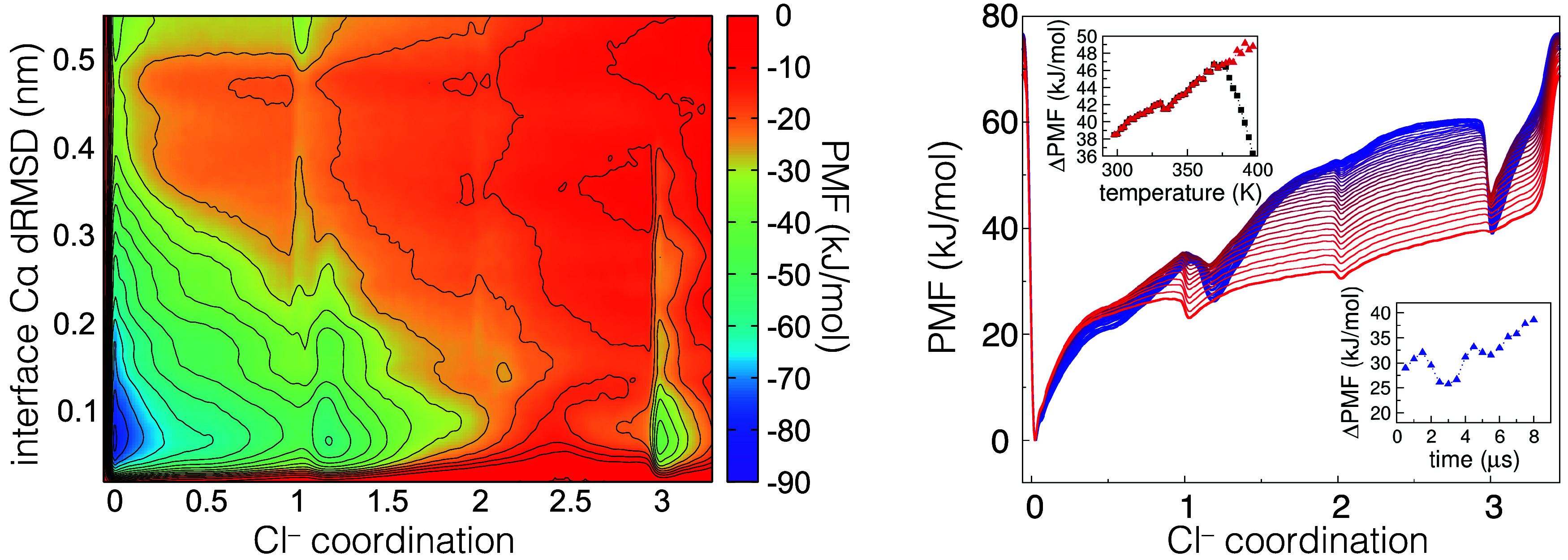
PMF (potential of mean force) for *2wpy* from the metadynamics simulations with the CHARMM36m+NBFIX force field. Left: 2D PMF for the 298 K replica. Right: 1D PMF as a function of Cl^–^ coordination for all of the replicas, colored from blue to red at increasing temperature. The upper left inset shows the ΔPMF between the minimum at Cl^–^ coordination ≃3 (bound state) and the one at ≃0 (unbound state) as a function of temperature (i.e., for the different replicas). The black squares show the values taken from the 1D curves, while the red triangles report the differences taking the basins from the 2D surface at Cα dRMSD < 0.2 nm. The lower right inset shows the same quantity for the 298 K replica as a function of the simulation time.

Nonetheless, the Cl^–^-bound state is still a metastable minimum, with a barrier of about 20 kJ/mol, in agreement with the persistence of Cl^–^ in the binding pocket observed in the previous plain MD simulations. There is clearly another metastable state at Cl^–^ coordination ≃1.25, which is actually more stable than the Cl^–^-bound state. This corresponds to configurations in which the binding site is empty but one Asn19 is contacted by an external Cl^–^. The number is larger than one because when a Cl^–^ is close to one ND2, the other two are in intermediate distances that contribute to the additional ≃0.25 of the CV.

We observe that, given the restraint on the maximum interface Cα dRMSD values, necessary to achieve sufficient sampling in the explored CV ranges, we do not observe full denaturation of the trimer at higher temperatures. Though the denatured state could reasonably be the most stable state, particularly for the high-temperature replicas, given the low stability of *2wpy*—its full exploration requires longer simulation times and a larger box accommodating the stretched helices, with subsequent increase in the number of replicas to span the same temperature range.

In the following, we switch to an independent method to calculate the free-energy of binding, namely, alchemical free-energy simulations. The metadynamics approach; however, explicitly simulates the entrance and exit of the Cl^–^ from the binding site. We report in Figures S9 and S10 of the SI the analysis of an unbinding event, showing how, during the exit of Cl^–^ from the binding site, one Asn19 keeps interacting with Cl^–^ and an additional transient interaction with Lys15 on the same helix is established. This helix undergoes a noticeable distortion during the extraction process (Figure S10), but the distortion is soon relieved after Cl^–^ separates from the coiled coil.

### Cl^–^-Binding Thermodynamics from Alchemical Free-Energy Simulations

To confirm the previous result on the Cl^–^ binding free energy in *2wpy* and compare it with the other coiled coils, we computed the Δ*G*_bind_ using an independent route — alchemical free-energy simulations by the double-decoupling method (eqs 2, 3, 4, 5, 6).^41^ Table 3 reports the results of this approach for the three TCCs, together with the various contributions associated with decoupling of Coulomb and van der Waals interactions and the establishment of the restraints on the Cl^–^ ion (Δ*G*_Cl →0,prot_^Coul^, Δ*G*_Cl →0,prot_^vdW^, and Δ*G*_prot_^restr^, respectively). The free-energy change associated with decoupling Cl^–^ from the protein environment (Δ*G*_Cl →0,prot_) is obtained by summing these terms and subtracting the analytically defined Δ*G*_vac_^restr^ = 21.3 kJ/mol (see eqs 4,5) (21.5 kJ/mol for *4dzk*). Finally, the Cl^–^ binding free energy is the difference between the decoupling of the Cl^–^ from the protein and from the solvent (the latter indicated with Δ*G*_Cl →0, sol_, and by definition given by −Δ*G*_hyd_).

**Table 3:** Summary of Δ*G* Values, in Kilojoules per Mole, from the Alchemical Free-Energy Simulations

system and settings^a^		runs	Δ*G*_Cl→0_^Coul^	Δ*G*_Cl→0_^vdW^	Δ*G*_prot_^restr^	Δ*G*_Cl→0_^prot^ ^b^	Δ*G*_bind_ ^c^
*2wpy*	cubic	4	393.7 (3.3)^d^	–19.5 (1.0)	2.8 (0.2)	355.7 (3.8)	29.8 (3.9)
	tr. oct.	3	393.8 (2.2)	–21.9 (1.1)	2.8 (0.2)	353.4 (2.3)	32.1 (2.4)
	tr. oct. (0 →Cl)^e^	3	394.5 (1.7)	–24.2 (1.1)	3.6 (0.4)	352.6 (1.1)	32.9 (1.2)
*2wpy*	tr. oct. H18^+^	3	403.1 (2.9)	–24.7 (1.3)	3.3 (0.2)	360.4 (2.9)	25.1 (3.0)
*2wpy*	tr. oct. (ff:a14sb)	2	361.0 (1.2)	–13.9 (1.1)	4.5 (0.5)	330.2 (1.2)	42.7 (1.3)
*2wpy*	tr. oct. (ff:a99sb-disp)	2	377.9 (1.7)	–18.5 (1.8)	5.3 (0.7)	343.3 (2.3)	31.2 (2.4)
*4dzk*	tr. oct.	2	402.4 (1.6)	–16.7 (0.7)	3.6 (0.1)	367.7 (1.6)	17.8 (1.7)
*4dzk*-cap	tr. oct.	2	401.7 (1.6)	–16.7 (0.7)	3.8 (0.2)	367.3 (1.6)	18.2 (1.7)
*1mof*	tr. oct.	3	445.3 (1.5)	–11.1 (2.2)	2.0 (0.2)	414.9 (3.2)	–29.4 (3.3)
	tr. oct. (0 →Cl)	2	440.0 (2.4)	–9.9 (1.4)	3.4 (1.8)	412.2 (2.7)	–26.7 (2.8)
*1mof*	tr. oct. open leash^f^	2	429.8 (3.2)	–17.8 (3.4)	2.8 (0.0)	393.6 (5.5)	–8.0 (5.6)
						Δ*G*_Cl→0_^sol^	
*water*		1	400.2 (0.1)	–14.7 (0.1)		385.5 (0.1)	
*water*	(ff:a14sb)	1	394.7 (0.1)	–21.9 (0.1)		372.9 (0.1)	
*water*	(ff:a99sb-disp)	1	397.7 (0.1)	–23.1 (0.2)		374.5 (0.2)	

a The force field is CHARMM36m with NBFIX corrections, unless indicated otherwise.

b Δ*G*_Cl→0_^prot^ = Δ*G*_Cl→0_^Coul^ + Δ*G*_Cl→0_^vdW^ + Δ*G*_prot_^restr^ – Δ*G*_vac_^restr^ where the last term is 21.3 kJ/mol for *2wpy* and *1mof*, and 21.5 kJ/mol for *4dzk*.

c For each protein Δ*G*_bind_ = Δ*G*_Cl→0_^sol^ – Δ*G*_Cl→0_^prot^ according to the force field used.

d Uncertainties in parentheses are calculated with the binning method.

e 0 →Cl values are reported with reversed sign for consistency.

f These runs start from the plain-MD *1mof* simulation (MD2_–Cl_) featuring a complete opening of one of the leashes.

The obtained Δ*G*_hyd_ for Cl^–^ is −385.5 (0.1) kJ/mol in the case of CHARMM36m with the NBFIX correction, in agreement with previous calculations^31^ (the so-called intrinsic Δ*G*_hyd_ is the appropriate one for the thermodynamic cycle used hereby). The same quantity in the case of the Amber14sb and Amber99sb-disp force fields is −372.9 (0.1) kJ/mol and −374.5 (0.2) kJ/mol, respectively.

The various rows for the same system in Table 3 refer to different settings, such as the kind of solvation box used (cubic or truncated octahedron), the force field used, the protonation state of the histidine residue H18 (neutral or protonated) in the case of *2wpy*, the capping treatment for *4dzk* and the leash configuration for *1mof*. The systems and settings are listed in Table 1, and the complete list of runs is reported in the Supporting Information (Tables S4, S5, and S6).

The values of Δ*G*_bind_ calculated with the alchemical approach for *2wpy* are 30–33 kJ/mol using CHARMM36m with NBFIX, and they are in good agreement with the value obtained from the replica-exchange metadynamics simulations. This agreement and the negligible difference between the calculations with different box types and sizes (cubic vs tr. oct.) point to a minor role of finite-size effects due to decoupling of charged species. Indeed, when counterions are used to neutralize the system, as in the present case, the corrections needed to extrapolate for the infinite system are limited in size (≲1 kJ/mol).^53^ The other two force fields also predict a largely unfavorable Cl^–^ binding in *2wpy*, even 10 kJ/mol higher with Amber14sb. The case of Amber99sb-disp, which uses a 4-site water model (TIP4P-D), falls within the same range of CHARMM36m with NBFIX. When the histidine residues of *2wpy* are protonated (H18^+^), the extra positive charges lower the Δ*G*_bind_ by ≃6 kJ/mol, which is; however, not a large enough drop to guarantee a favorable binding.

We applied the same approach to the other two TCCs. Cl^–^ binding results are unfavorable also in the case of *4dzk*, though by a smaller degree (Δ*G*_bind_ ≃ 18 kJ/mol). We checked that a different treatment of capping has no effect on the Δ*G*_bind_ (*4dzk*-cap has neutral termini while *4dzk* has charged termini; see Methods section). By contrast, in the case of *1mof* the Δ*G*_bind_ is largely negative (between −27 and −29 kJ/mol), pointing to a rather strong binding. However, the strength of this binding is dependent on the presence of an intact leash layer. Indeed, when an open-leash configuration is used as the starting structure for the alchemical run, the Δ*G*_bind_ increases to −8 kJ/mol. This remarkable increase hints at a structural role of the leash in modulating the properties of the binding site and is key to understanding why *1mof* shows such a different Δ*G*_bind_ with respect to the other systems.

#### Asn Flipping and Differences among the Runs

Before addressing the differences among the three systems, we focused on the differences within the various runs of the same system. We observe that, in particular for *1mof*, there is a sizable 5.3 kJ/mol difference between the Δ*G* ^Coul^ of Cl→ 0 and 0 →Cl, which is partially compensated by an opposite change in the vdW. Figure 8 shows the average values and distributions of ∂ *H*/∂λ for each replica (i.e., value of λ) in the Coulomb decoupling step. The difference in Δ*G* ^Coul^ occurs mostly in the ranges 0.6–0.8 and 0.9–1 of λ_Coul_. The distribution of ∂*H*/∂λ in the simulations shows a biphasic behavior around 0.7/0.8. Inspection of the corresponding MD trajectories shows flipping of the Asn side chains from a configuration in which all the NH_2_ amino groups point to the center (i.e., to the Cl^–^), to configurations in which one of the Asn side chain has a flipped amide group (180 degree rotation around χ_2_) or adopts another rotamer around χ_1_, both cases resulting in the amide oxygen atom pointing toward the center. Clearly, as the charge on the Cl^–^ decreases (it is 0 at λ_Coul_ = 1), the former configuration becomes less favorable. In general, the rather large uncertainties in our calculated Δ*G* ^Coul^ also in the case of *2wpy* and *4dzk* can be, for a large part, attributed to different sampling of these two situations.

**Figure 8:**
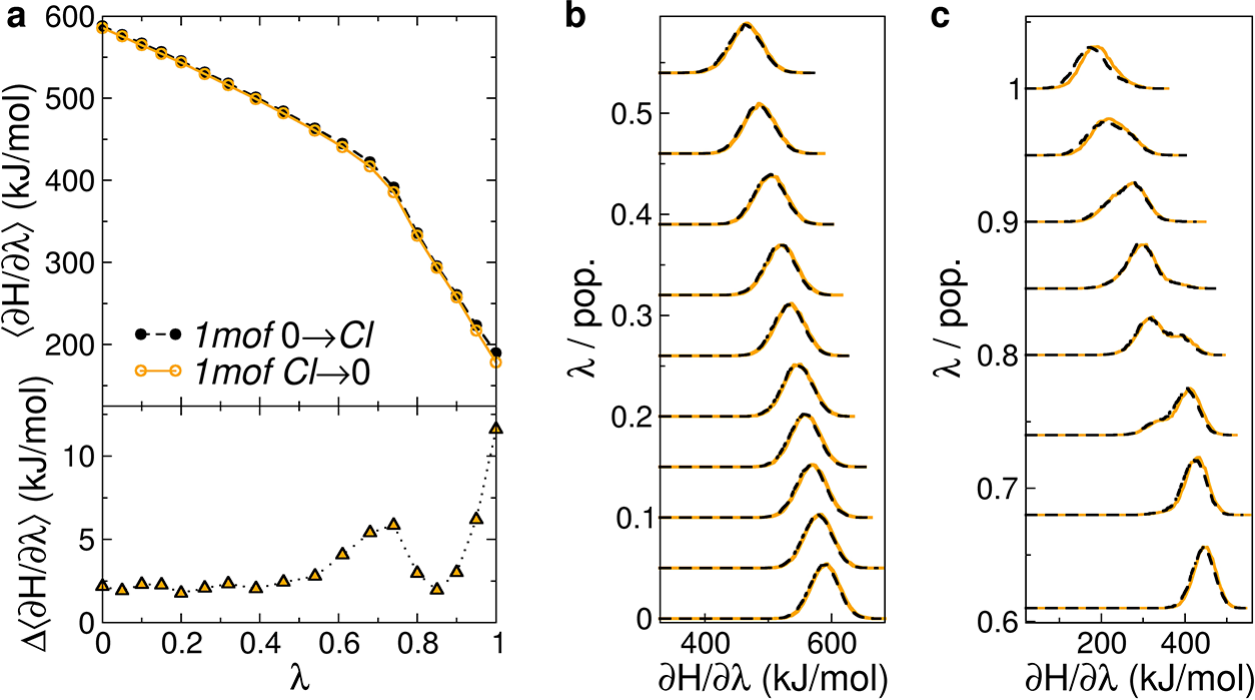
∂*H*/∂λ in the Coulomb decoupling step from representative runs of the Cl→ 0 (orange circles and solid lines) and 0 →Cl (black circles and dashed lines) processes referred to equivalent λ values. Panel (a) reports the average values for the two cases (top) and their difference (bottom). Panels (b, c) show the distribution (pop.) of ∂*H*/∂λ for each replica, translated according to the corresponding (equivalent) λ value.

There are, however, additional factors that contribute to the hysteresis in *1mof*, namely different water occupation of the binding site for the decoupled state in the two processes (Cl→ 0 and 0 →Cl) and leash mobility. The interplay between leash mobility and Cl^–^ binding thermodynamics is addressed at the end of this section. Regarding the former factor, in Cl→ 0 ≃ 70% of the snapshots show a water molecule in the Cl^–^ binding site, while the percentage is ≃88% in 0 →Cl. As revealed in the plain MD simulations, whereas *2wpy* and *4dzk* are rather permeable to water molecules, the core of *1mof* is more rigid, and the Cl^–^ binding site is protected from the solvent by the additional protein layer formed by the C-terminal leash of each monomer. Clearly, a partial occupation in the decoupled state, potentially due to incomplete sampling, may result in energetically unfavorable empty cavities, artificially raising the free energy of the Cl^–^ unbound state.

To address this issue, we performed a thermodynamic cycle transforming the Cl^–^ into a water molecule (Cl→W process). We verified that this internal water molecule stays in the binding site for each of the replicas, so that no restraint is needed to avoid migration in the bulk solvent, and only the Coulomb and van der Waals components need to be calculated. As reported in Table 4, the resulting Δ*G*_bind_ ≃ −25 kJ/mol (see eq 6) is in line with the values predicted from the Cl→ 0 and 0 →Cl transformations and actually closer to the latter in line with the higher water occupancy of the binding site in the decoupled state. The same approach was not feasible for the other two TCCs, because the water molecule easily migrates into the bulk.

**Table 4:** Δ*G* Values for the Cl →W Transformation in *1mof*

settings	runs	Δ*G*_Cl→W_^Coul^	Δ*G*_Cl →W_^vdW^	Δ*G*_Cl→W_	Δ*G*_bind_ ^a^
*1mof* tr. oct.	3	448.8 (3.2)	–55.8 (0.7)	393.1 (3.7)	–25.1 (4.0)
*water* tr. oct.	1	484.1 (1.2)	–126.1 (0.7)	358.0 (1.4)	

a Δ*G*_bind_ is calculated using eq 6.

To further clarify the presence and stability of water molecules within the binding site, both in the presence and absence of the central Cl^–^, we computed the corresponding Δ*G*_bind_ values for a water molecule using the same simulation protocol employed for the Cl→ 0 alchemical transformations. The results, reported in Table 5, confirm that in *1mof*, a water molecule is stably bound in the binding site in the absence of Cl^–^. By contrast, the presence of additional water molecules adjacent to the central ion (a feature observed in both *2wpy* and *4dzk*) is predicted to be thermodynamically unfavorable.

**Table 5:** Δ*G* Values for the W→0 Transformation in *1mof*

settings	Δ*G*_W→0_^Coul^	Δ*G*_W→0_^vdW^	Δ*G*_W→0_^restr^	Δ*G*_W→0_	Δ*G*_bind_ ^a^
*1mof*–CL tr. oct.	43.4 (0.7)	3.3 (2.2)	3.7 (0.1)	39.0 (2.8)	–12.6 (2.9)
*1mof*+CL tr. oct.	16.0 (0.6)	–4.7 (0.2)	1.6 (0.1)	4.0 (0.9)	22.4 (1.0)
*water* tr. oct.	35.3 (0.1)	–8.8 (0.1)	26.4 (0.1)		

a Δ*G*_W→0_^prot^ = Δ*G*_W→0_^Coul^ + Δ*G*_W→0_^vdW^ + Δ*G*_prot_^restr^ – Δ*G*_vac_^restr^ where the last term is 11.4 kJ/mol for *1mof*–CL, and 8.9 kJ/mol for 1mof+CL, respectively.

In the case of *1mof*, we also calculated the difference in Δ*G*_bind_ for Cl^–^ and F^–^ by transforming the ion within the protein and in the water box. In the protein Δ*G*_Cl→F,prot_ = −69.5(0.1) kJ/mol and in the solvent box Δ*G*_Cl →F,solv_ = −108.1(0.1) kJ/mol, resulting in *Δ*Δ*G*_bind_ = 38.6 (0.1) kJ/mol. This would imply that the Δ*G*_bind_ for F^–^ would be positive, at around 13 kJ/mol. Such a value is in agreement with the experimental observation that the melting temperature of *1mof* was unaffected by the presence of F^–^ salts in solution.^11^

#### Origin of Different Cl^–^-Binding Thermodynamics

To understand the determinants of the markedly different Δ*G*_bind_ of *1mof* vs the other two TCCs, we compare in Figure 9 the ⟨∂*H*/∂λ⟩ profiles for each system. The Coulomb term is by far the largest and accounts for most of the variation. The ∂*H*/∂λ_Coul_ is simply the opposite of the average electrostatic energy of the fully charged Cl^–^, evaluated in the various ensembles of configurations generated by the mixed potential energy at each λ_Coul_ value—that is, with the charge on the Cl^–^ gradually reduced to 0, while keeping the LJ parameters unmodified and the restraints turned on. A rigorous comparison of the electrostatic energy can only be made after accounting for finite-size corrections, which differ across the systems due to different protein charges and box shapes and sizes. Nonetheless, some qualitative trends can be observed. At λ_Coul_ = 0 the ⟨∂*H*/∂λ_Coul_⟩ value for *1mof* is between that of *2wpy* and *4dzk*, hinting at a similar average electrostatic energy of the Cl^–^ in the bound state, i.e., the binding site geometry does not result in markedly different electrostatics in the three systems (the value in water is higher, reflecting a better coordination of the ion with respect to the protein binding site). The main difference emerges in the latter part of Coulombic decoupling. In this λ_Coul_ range, the *1mof* profile departs from the otherwise similar trends of *2wpy* and *4dzk*, showing higher ⟨∂*H*/∂λ⟩ values. This indicates that the binding site in *1mof* maintains, on average, a geometry that remains electrostatically favorable to the charged Cl^–^ even when its charge is reduced to zero. We attribute this behavior to the higher rigidity of the *1mof* core, which does not allow the binding site to relax its geometry in response to the progressive neutralization of the ion, as in the case of the other two TCCs. Indeed, when the TCC core of *1mof* is destabilized because of leash opening (*1mof* open leash), the behavior at λ_Coul_ above 0.6 differs from that of intact *1mof*, resulting in lower ⟨∂*H*/∂λ⟩ values.

**Figure 9:**
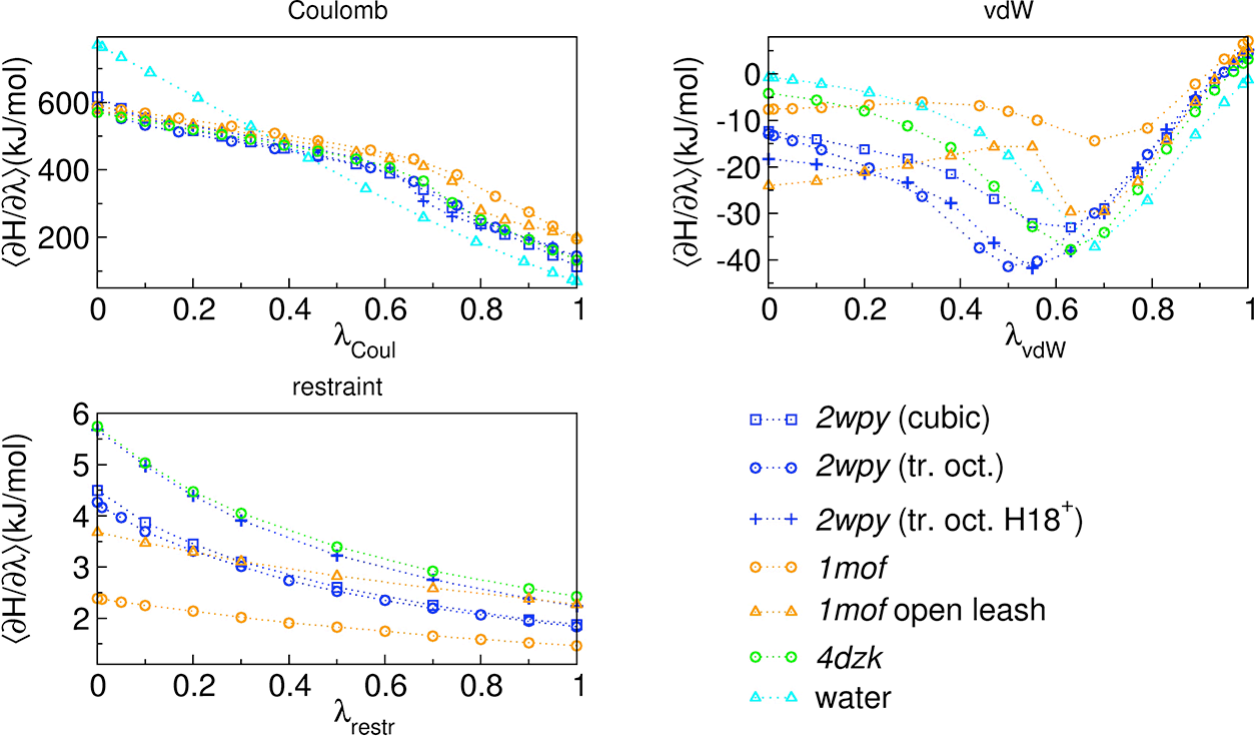
⟨∂*H*/∂λ⟩ profiles for the various alchemical contributions—restraint, Coulomb, and van der Waals (vdW) as a function of the coupling parameter λ. At λ = 0, the interaction is fully present, while at λ = 1 it has been completely turned off (decoupled). Each data set is from a representative run of selected rows in Table 3.

The different trends for the vdW contribution do not lend themselves to a straightforward interpretation due to the presence of both the attractive and repulsive parts in the LJ interaction and the use of the soft-core interactions. Overall, however, the vdW term also gives *1mof* an advantageous contribution to Cl^–^ binding, of up to 10 kJ/mol over *2wpy*. Finally, though the restraint contribution is much smaller, its trends, with a significantly lower value for *1mof* at λ_restr_ = 0, again reflect the effect of the higher rigidity of the *1mof* binding site, which limits the fluctuations in the position of Cl^–^, as previously observed from the RMSF analysis (Figure 3, bottom left panel). Again, this rigidity is partially lost in the configuration with an open leash.

### Variation of *R*_0_, Water Occupation, and Leash Mobility during the Alchemical Transformations

In selected alchemical runs, we calculated the local super helical radius (*R*_0_) during each λ window. Consistent with the treatment for the plain MD simulations, the main-chain of Asn@*d*-containing heptad was averaged during the 10–50 ns time interval, and the coil-coiled parameters were obtained through the described fitting procedure. Water occupancy was also calculated by counting the percentage of trajectory frames with one or more water molecules within a 0.4-nm cutoff of the central chloride. The results are shown in Figure 10.

**Figure 10:**
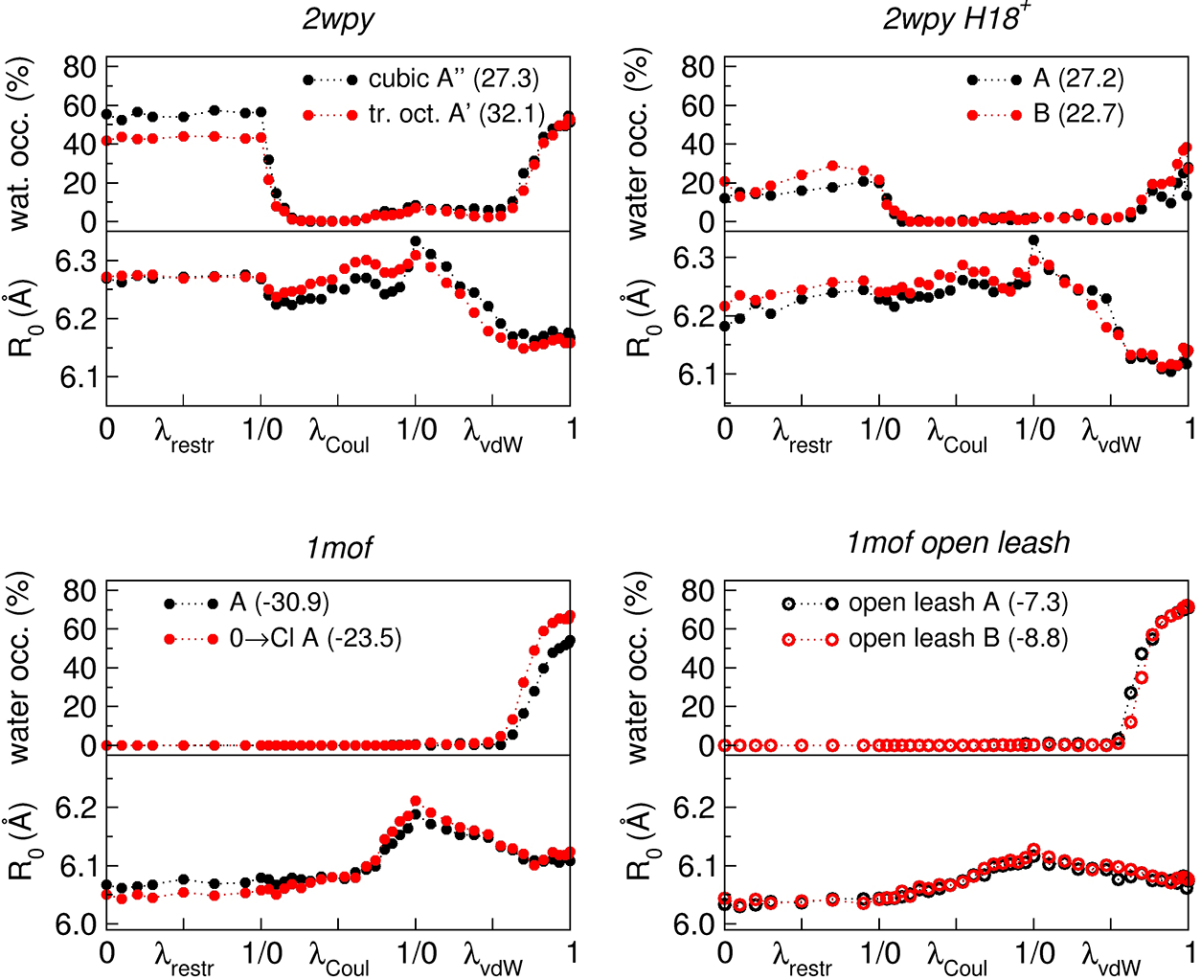
Water occupancy of the Cl^–^ binding site and superhelical radius *R*_0_ during the alchemical transformations for selected runs. The *x*-axis reports the value of λ for each of the three steps (restraint, Coulomb, and van der Waals). λ_restr_ = 0 corresponds to the fully interacting Cl^–^ with no restraint (i.e., the Cl^–^ bound state), while λ_vdW_ = 1 corresponds to the fully decoupled dummy particle (i.e., the Cl^–^ unbound state). The plot legends indicate the corresponding alchemical run (see Tables S4 and S5), with the resulting Δ*G*_bind_ values reported in parentheses (in kJ/mol). Unless explicitly stated, the data refer to Cl→0 runs.

Water occupancy exhibits complex behavior in *2wpy*. It starts at approximately 50% (15–20% in the H18^+^ case), consistent with plain MD simulations. During the Coulomb decoupling step, water occupancy drops rapidly to zero even for small reductions in the particle charge. Water then re-enters the binding site during the Lennard–Jones decoupling (λ_vdW_ values between 0.5 and 1), reaching occupancies higher than those observed in the plain MD run. Notably, in this final segment, where water occupancy changes most significantly, *R*_0_ remains essentially constant. This suggests a compensation between the increasing presence of water molecules and the progressive removal of the Lennard–Jones interactions of the ion. In this final stage of the alchemical transformation, *1mof* displays qualitatively similar behavior, characterized by a high, though not complete, water occupancy of the binding site.

Regarding the behavior of *R*_0_, all systems exhibit a pronounced peak at the end of the Coulomb decoupling step, where the charge of the central particle is neutralized, while its full Lennard–Jones interactions are retained. This effect is particularly marked in intact *1mof*, whereas it is less pronounced when the leash is open. The initial and final states of the alchemical transformations reproduce the *R*_0_ trends observed in the plain MD simulations, although the variations are generally reduced. In *2wpy*, the resulting Δ*R*_0_ is 0.10 Å, compared with the 0.16–0.17 Å variation obtained from plain MD. With the protonated histidine (H18^+^) it is 0.07/0.08 Å. In *1mof*, the Δ*R*_0_ values are −0.04 Å (run A) and −0.07 Å (run 0 →Cl A), in agreement with the −0.07 Å change observed in the corresponding plain MD simulations. When the leash is open, the variation is around −0.04 Å, and the intermediate states sampled during the transformation display a less marked structural response.

We finally monitored leash fluctuations in the initial and final states of the alchemical transformations for *1mof* (Figure 11). In the simulations with a “closed” leash, a range of conformational behaviors are sampled, with individual chains within the same run often displaying different mobilities. Because leash rearrangements occur on timescales longer than those accessed during the alchemical transformations, the initial configuration leaves a persistent imprint on the dynamics (for example, the higher leash-ter dRMSD of chain B, with respect to A and C, in the Cl→ 0 run). There are indications of enhanced hinge fluctuations in the decoupled 0 state, most notably in the 0 →Cl run, which also exhibits increased mobility in the leash-ter region. This is another factor, besides water occupation of the binding site, at the basis of the different calculated Δ*G*_bind_ values in the Cl→ 0 and 0 →Cl runs. Conversely, in the open-leash simulations, the Cl^–^-bound state occasionally undergoes transitions toward a closed-leash configuration. While these observations suggest potentially meaningful correlations, fully characterizing them would require alchemical simulations extending to even longer timescales than those considered in the present study.

**Figure 11:**
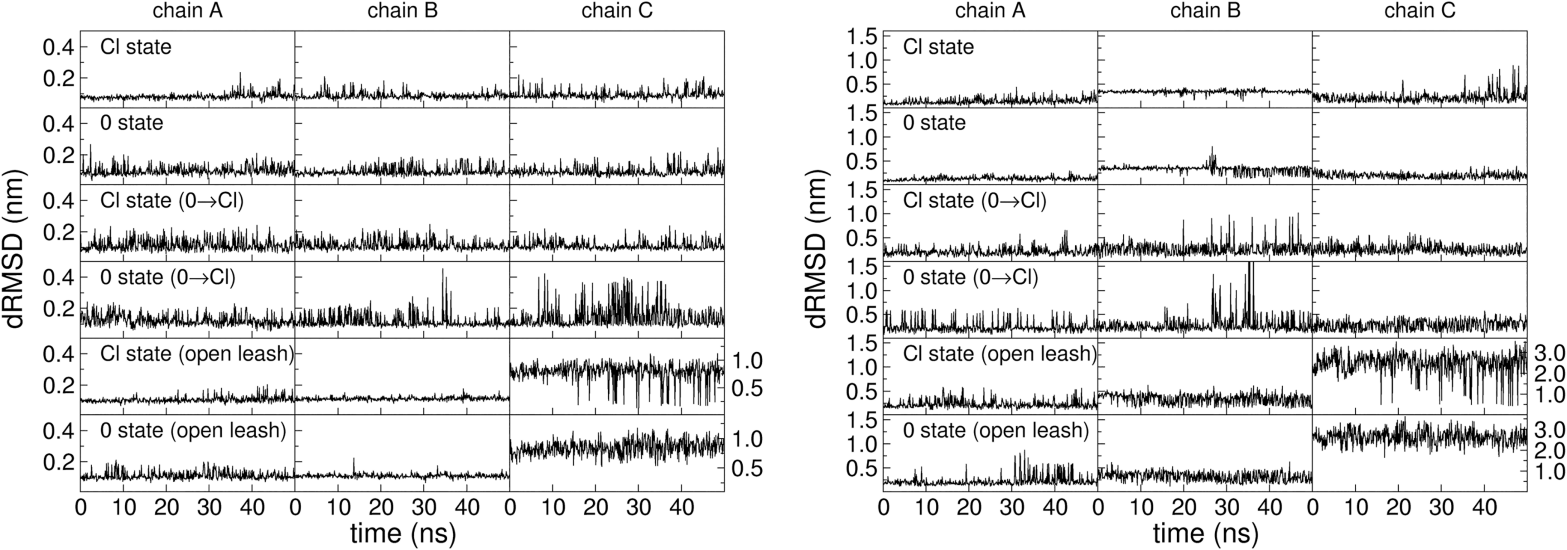
Left panel: RMSD to the X-ray structure of interatomic distances (dRMSD) at the interface between the hinge region (residues 80_*i*_–93_*i*_) and the coiled-coil core in *1mof* during the alchemical transformations. The dRMSD is shown for the start and end states (“Cl state” and “0 state”) for the Cl→ 0 A run (first and second plots from top), the 0 →Cl A run (middle) and for the Cl→ 0 open leash A run (bottom). For detail about the calculation of the dRMSD values see the caption of Figure S5. Right panel: same as left but for the leash-ter region (residues 94_*i*_–98_*i*_). For chain C of the open-leash run, a different *y*-axis is used, given the higher fluctuations.

## Discussion and Conclusions

Our investigation into halide-binding trimeric coiled coils started by exploring the mechanisms of Cl^–^-induced stabilization of these structures, through an MD simulation approach. As a starting point, we considered the case of *2wpy*, a metastable variant of GNC4-p1. For this system, circular dichroism measurements showed a steep decrease in ellipticity (indicative of increased α-helical content) over the first few hundred millimolar NaCl, followed by a more gradual decline up to 2 M.^10^ We therefore asked whether MD simulations of *2wpy* in the absence of Cl^–^ were able to capture the transition into a partially or even completely disordered structure.

This is definitely a challenging task for MD simulations due to the limitations of standard biomolecular force fields on one hand to balance the description of folded and disordered structures,^29,54,55^ and on the other to properly describe protein-ion interactions – an issue that is particularly acute for highly polarizable anions such as chloride.^31,56^ With respect to the first challenge, it is worth noting that a recent MD study by Notari et al.^23^ demonstrated that the stability and conformational switching of coiled-coil assemblies can nonetheless be correctly predicted using enhanced-sampling metadynamics in combination with the Amber ff96 force field and an implicit solvent model.

Against this background, we were, however, surprised to find that, in addition to the absence of any chloride stabilization effect, the binding of the anion to the Asn-triad binding pocket was highly unstable in *2wpy*. This instability was present even when including the latest corrections (via pair-specific LJ parameters) to Cl^–^-protein interactions, which were shown to mitigate some of the deficiencies of non-pair-specific LJ terms.^31^ We first quantified this instability, showing a largely positive Δ*G*_bind_ ≃30 kJ/mol, both by potential of mean force calculations via replica-exchange metadynamics and by alchemical transformations. The consistency between these two independent approaches guarantees that the calculated free energies are broadly converged, which is a nontrivial achievement given the need to account for the overall flexibility of the system and, in particular, the high mobility of the asparagine side chains in the unbound state. We observed that the instability of Cl^–^ binding is slightly moderated when His18 in the sequence is protonated, leading to a Δ*G*_bind_ ≃25 kJ/mol.

We then extended the analysis to other TCC containing the same Cl^–^-binding asparagine triad. *4dzk*, the synthetic TCC-forming peptide by Woolfson and co-workers^27^ gave similarly, if less dramatic, unstable Cl^–^ binding (Δ*G*_bind_ ≃18 kJ/mol). By contrast, the other system, *1mof* from a retroviral transmembrane protein,^11^ exhibited a remarkably favorable binding (Δ*G*_bind_ between −30 and −25 kJ/mol). *1mof* contains a TCC core, which is similar in size to the other two systems but features the additional C-terminal leash characteristic of these retroviral fusion-core fragments. This addition seemingly results in a more rigid binding-site structure in the MD simulations, and is at the basis of the prediction of a remarkably more stable binding. In keeping with this interpretation, when one of the leashes becomes fully detached from the coiled-coil core, the predicted Δ*G*_bind_ is markedly reduced in absolute value, increasing to approximately −8 kJ/mol. This observation points to a coupling between the leash conformational stability and chloride-binding thermodynamics. Although from our simulations we could not establish a systematic correlation between chloride removal and enhanced leash mobility, increased flexibility of the leash in the unbound state could nonetheless contribute to a reduced binding affinity and merits further computational and experimental investigation.

An interesting association emerges from the plain MD simulations of the Cl^–^-bound and unbound states. In addition to highlighting the different flexibilities of the three TCCs, the simulations reveal subtle but significantly distinct variations of the coiled-coil architecture near the binding site: upon chloride removal, the local superhelical radius *R*_0_ gets shorter in *2wpy*, remains essentially unchanged in *4dzk*, and increases in *1mof*, broadly reflecting the trend in the calculated Δ*G*_bind_, as shown in Figure 12. In *2wpy*, chloride removal locally tightens the coiled coil, possibly because the overall flexibility allows for inward relaxation of the binding cavity. The opposite happens in *1mof*, where the more rigid structure of the core prevents such a relaxation, and the cavity, whether empty or occupied by a water molecule, enforces a network of interactions that induces a local expansion of the structure.

**Figure 12:**
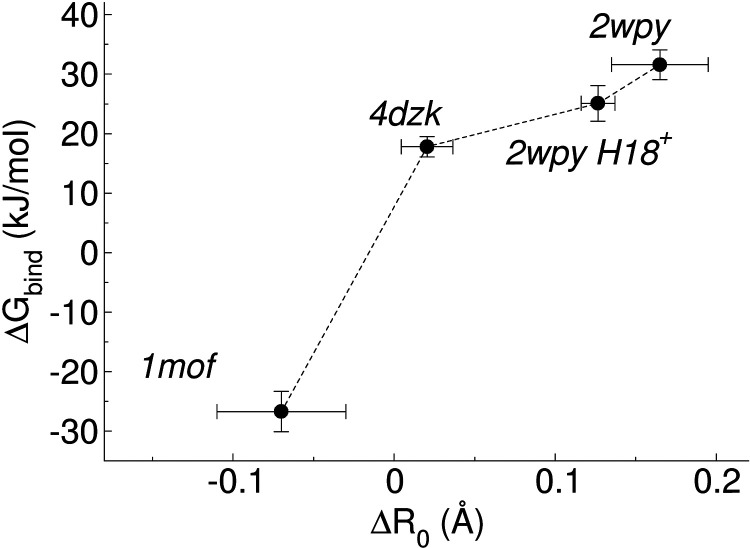
Cl^–^ binding free energy (Δ*G*_bind_) vs the variation of local superhelical radius Δ*R*_0_ between the Cl^–^ bound and unbound states for the set of investigated TCC. The values for Δ*G*_bind_ are averages from Tables 3 and 4 (the open leash runs were excluded from the *1mof* average), whereas the Δ*R*_0_ values are from Table 2, and are obtained by Crick-parameters analysis of the averaged structure (Asn@*d*-containing heptad only) during the plain MD simulations.

Notably, analogous changes in *R*_0_ are not systematically reproduced along the alchemical transformations, suggesting that there is no direct causal relationship between the observed variations in the superhelical geometry and the distinct thermodynamics of chloride binding. Nevertheless, within the MD description, this correlation points to an internal consistency between the local structural response of the coiled coil and the computed binding thermodynamics. If confirmed across a broader set of TCCs, this correlation would suggest that variations in *R*_0_ upon ion removal may encode information about relative binding thermodynamics, potentially providing an inexpensive structural indicator of binding affinity in these systems. At the same time, partial loss of symmetry in the unbound state indicates that other anisotropic structural descriptors, such as helix–helix distances and interfacial packing metrics, may provide a more complete characterization of the structural response to ion removal.

Direct measurements of the Cl^–^ binding affinity in these coiled coils are, unfortunately, not available for direct comparison with our results. A rough estimate can nevertheless be inferred from the circular dichroism experiments on *2wpy* (see above), which suggest a dissociation constant on the order of 0.1–1 M. This corresponds to a Δ*G*_bind_ between 0 and −6 kJ/mol, at least 20 kJ/mol more favorable than our computed value for the H18^+^ case. For the other two TCCs, experimental evidence points to stable chloride binding under the investigated conditions of the peptide concentration, temperature, and ionic strength. In addition to the presence of a bound chloride in the X-ray structures (in all three TCCs), the increase in melting temperature *T*_m_ for *1mof* at a NaCl concentration of 5 mM^11^ can be explained only by a specific binding of Cl^–^ to the Asn triad, because it is absent in the Asn-substituted variant. The interaction is also halide specific, as F^–^ salts—unlike NaCl—do not stabilize the fold.^11^ In agreement with this observed behavior, our simulations do predict the unfavorable binding of the fluoride anion.

In relative terms, although the thermodynamics of Cl^–^-binding is presumably different in these systems, an overall *Δ* Δ*G*_bind_ of more than 50 kJ/mol would correspond to dissociation constants differing by several orders of magnitude between *1mof* and the other two TCCs. Assuming a *K*_D_ of 1 M for *2wpy*, this would imply approximate *K*_D_ values of 50 mM for *4dzk* and in the nanomolar range for *1mof* (though in this case, the leash mobility discussed above may reduce, but not abolish, the difference). In the absence of experimental values of chloride-binding affinities in these systems, we can envision two limiting scenarios. In the first, the real Δ*G*_bind_ values are similar across the three systems. From our analysis, the difference in the calculated Δ*G*_bind_ values is mostly due to differences in the Cl^–^-unbound state, such as dilation/restriction of the binding site region and water occupancy. Under this scenario, the dominant source of error would lie in the force-field treatment of subtle protein–protein and protein-water interactions rather than in the direct interactions between chloride and the binding site. In the second scenario, the real Δ*G*_bind_ shows the same variation as the calculated values, implying that the relative binding thermodynamics (*Δ*Δ*G*_bind_) are well predicted. In this case, refining the interactions of chloride with polar and branched hydrophobic amino acids may uniformly shift the absolute binding free energies, bringing the computed Δ*G*_*bind*_ into closer agreement with the experiment. This latter possibility would benefit from comparison with simulations employing polarizable force fields and hybrid QM/MM approaches, which could help reveal how ion polarization impacts specific ion–protein interactions.

Our results highlight the need for further experimental characterization of this class of coiled coils to better understand halide-induced stabilization and the thermodynamics of chloride binding. At the same time, by revealing that nonpolarizable force fields fail to capture halide interactions in these systems, they identify a class of relatively simple protein structures that could serve as benchmarks for improving the description of anion–biomolecule interactions and/or the balance between bound and unbound states.

## Supplementary Material



## Data Availability

Simulation input files, PLUMED input scripts, analysis scripts and representative trajectories for the chloride-binding calculations in the three trimeric coiled coils (PDB IDs: *2wpy*, *4dzk*, *1mof*) are available on Zenodo (https://zenodo.org/records/17441543). Molecular dynamics simulations were performed using GROMACS (v. 2022 and 2024) with various force fields (mainly CHARMM36m, but also Amber14SB, Amber99SB-disp, and CHARMM22* in selected cases), and metadynamics calculations employed PLUMED 2.8.

## Notes

Additional analysis of plain MD simulations (Figures S1–S7); generalized Crick parameters for the examined coiled coils (Table S1–S3 and Figure S8); chloride unbinding event during a biased simulation (Figures S9 and S10); tabulated Δ*G* values for each MD run (Tables S4–S6) (PDF)
