# Supplementary material for: Chloride Binding in Trimeric Coiled Coils: Free Energy and Structural Determinants from Molecular Simulations
Source: J Chem Inf Model. 2026 Jan 30;66(4):2311–27. doi: 10.1021/acs.jcim.5c02565 (PMC12933892; doi:10.1021/acs.jcim.5c02565)
Supplement: Supplementary file 1 [file ci5c02565_si_001.pdf]

Chloride binding in trimeric Coiled Coils: Free Energy and Structural Determinants from Molecular Simulations Supporting Information

Riccardo Nifosí and Luca Bellucci*

NEST, CNR – Istituto Nanoscienze and Scuola Normale Superiore, Pisa, Italy

E-mail: riccardo.nifosi@cnr.it

References

(1) Grigoryan, G.; Degrado, W. F. Probing designability via a generalized model of helical bundle geometry. *Journal of Molecular Biology* **2011**, *405*, 1079–1100, Publisher: Elsevier Ltd.

(2) Bennett, C. H. Efficient estimation of free energy differences from Monte Carlo data. *Journal of Computational Physics* **1976**, *22*, 245–268.

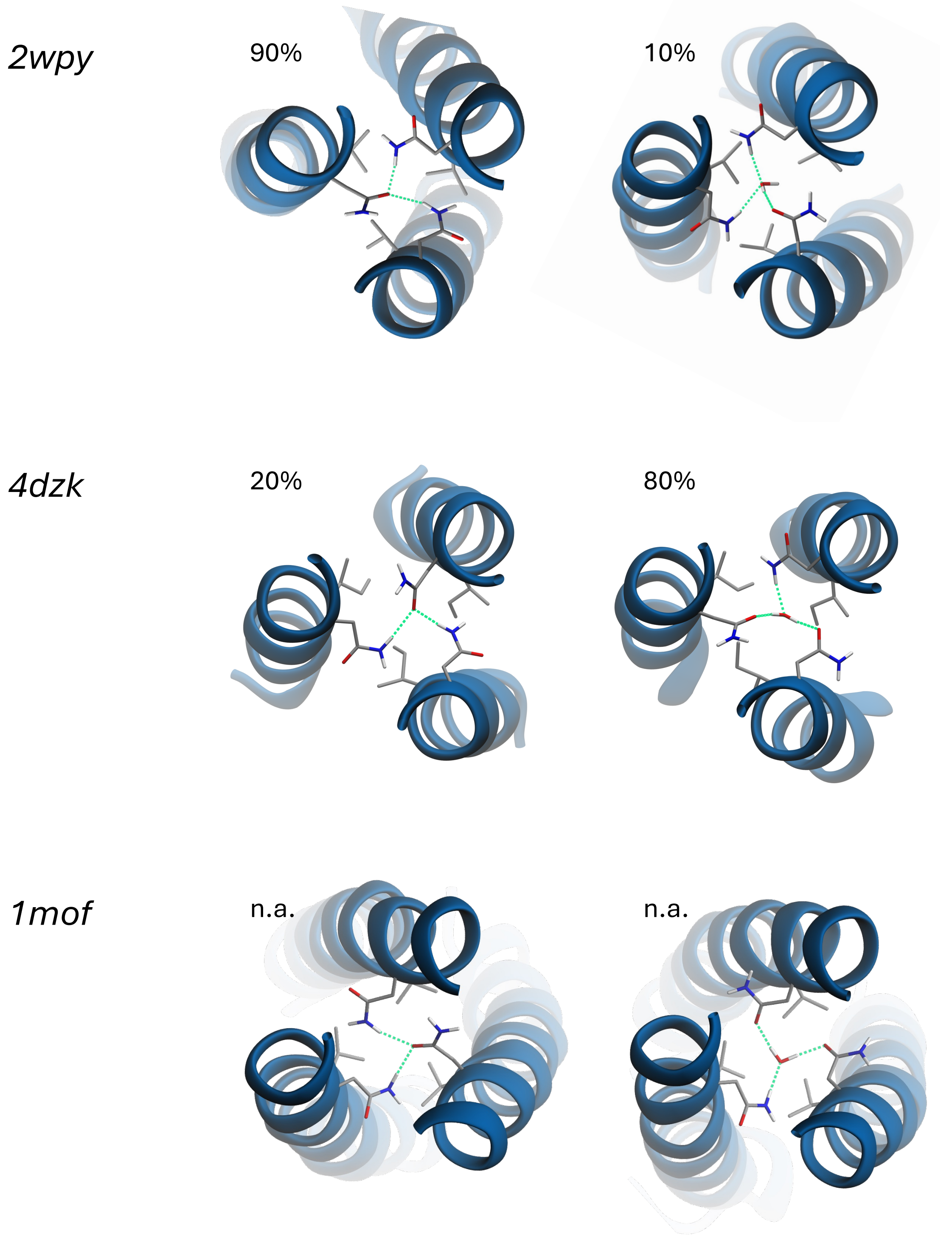

Figure S1: For each of the three TCC in the Cl$^-$-unbound state: the left and right panels show one representative snapshot in the absence and in the presence of the water molecule (in the case of *1mof* from two separate MD simulations, see main text). The percentages above the panels indicate the occurrence of each situation (presence or absence of a water molecule) during the simulation. In *1mof* there is little or no water exchange so the analysis of the occurrence was not feasible.

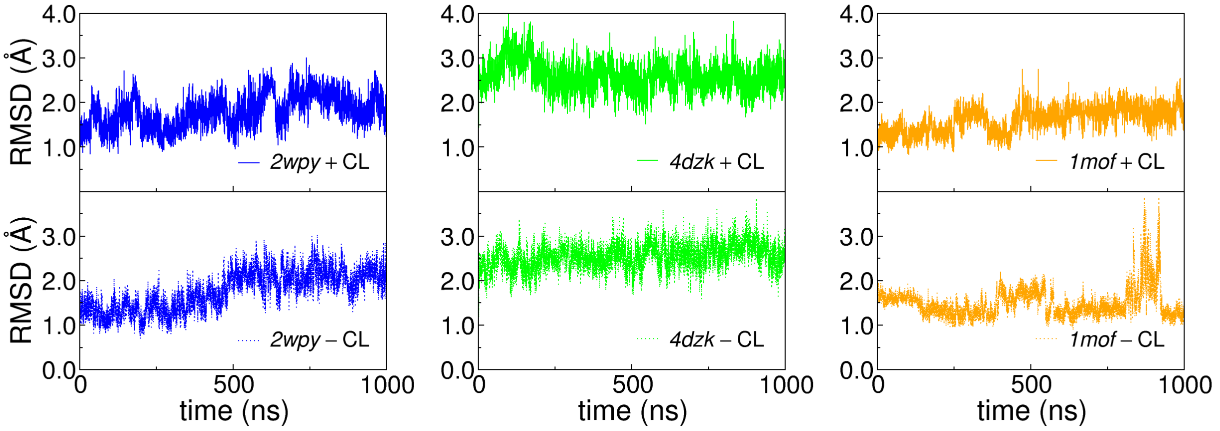

Figure S2: Root mean square distance (RMSD) from the starting X-ray structure with (+ CL, upper panels) and without (− CL, lower panels), during the simulations with the CHARMM36m+NBFIX force field. The RMSD are calculated for the atoms in the backbone. As discussed in the main text the spike in the *1mof* − CL run is due to partial (though reversible) unfolding of one of the C-terminal leashes.

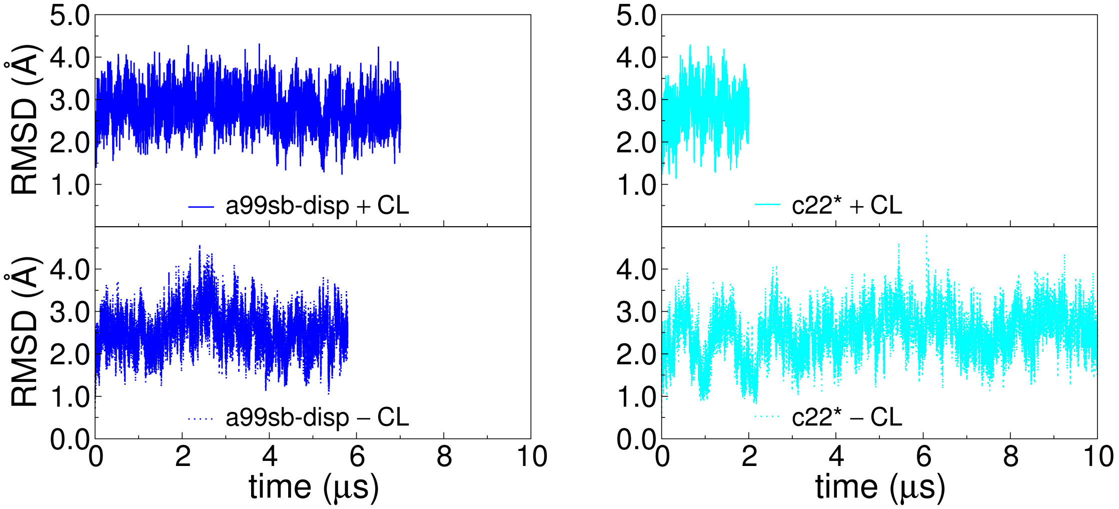

Figure S3: Root mean square distance (RMSD) from the starting X-ray structure with (upper panels) and without (lower panels), during the simulations of *1mof* with different force fields (amber99sb-disp left and CHARMM22* right). The RMSD are calculated for the atoms in the backbone. In the c22*–Cl simulation, the chloride ion was initially placed in the binding pocket but dissociated within the first 20 ns. Similar unbinding events were also observed in other runs with CHARMM22* (not shown).

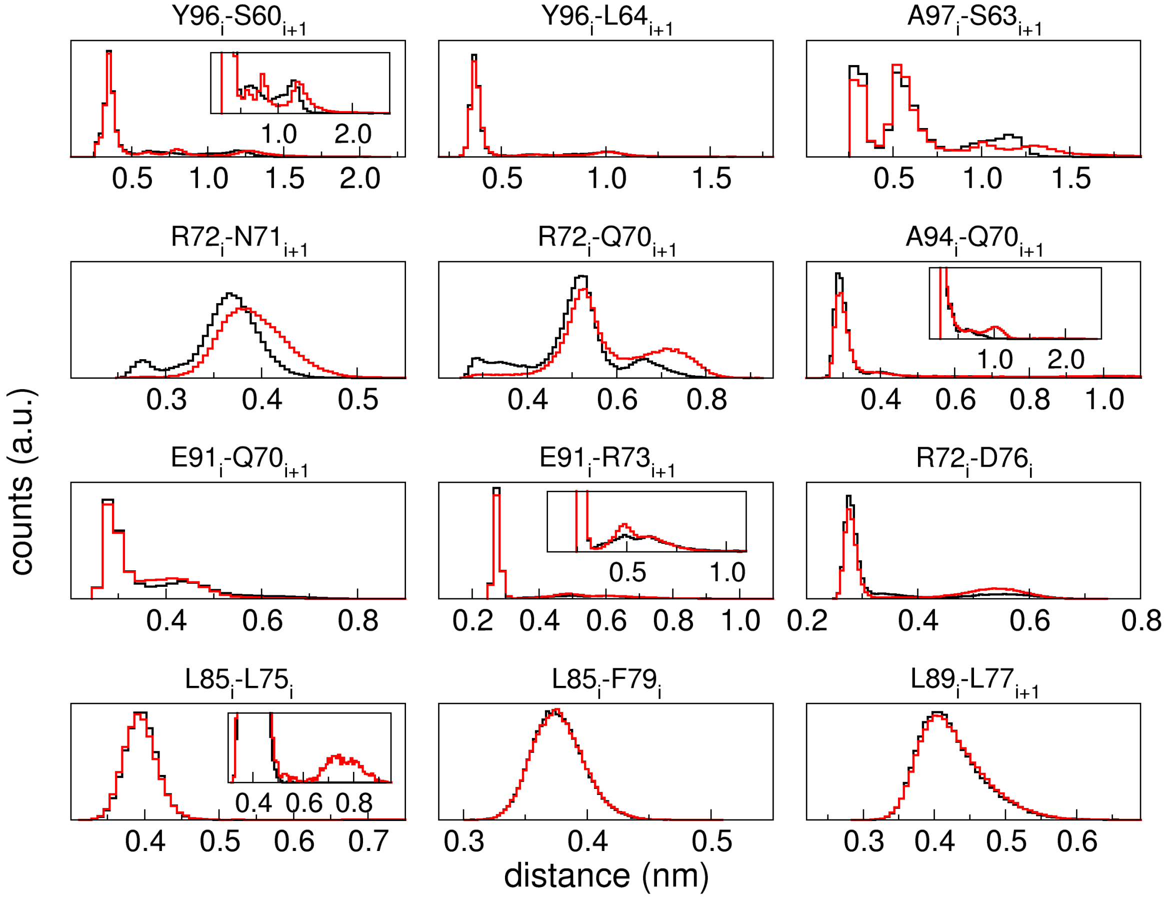

Figure S4: Histograms of minimum distances between residue pairs at the interface between leash and coiled-coil core. The black and red lines correspond to Cl^--bound and unbound simulations respectively. The histograms were computed by pooling all MD trajectories and considering only the second half of each simulation.

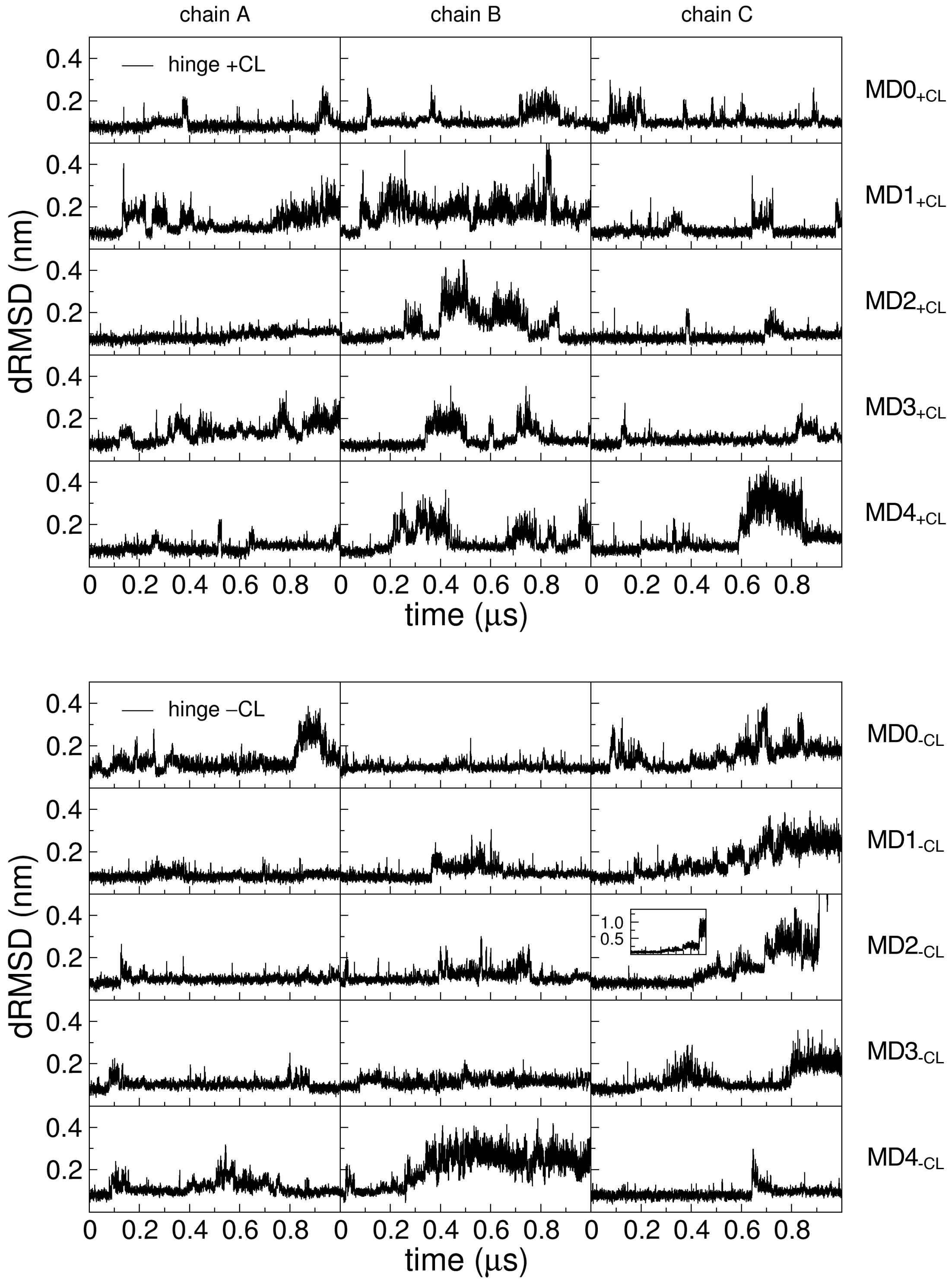

Figure S5: RMSD for interatomic distances (dRMSD) defining the interface between the hinge region (residues 80_i–93_i) and the coiled-coil core in *1mof*. The dRMSD is shown for five independent MD simulations with (top) and without (bottom) Cl^- bound at the central site. The dRMSD was computed over all non-H atom pairs that are within 0.5 nm in the X-ray structure, with one atom belonging to the hinge region and the other to the interface residues of the coiled-coil core (72_i, 75_i, 76_i, 79_i, 70_{i+1}, 71_{i+1}, 73_{i+1}, and 77_{i+1}). The full dRMSD trace for chain C in $\mathrm{MD2}_{-\mathrm{CL}}$ is shown in the inset.

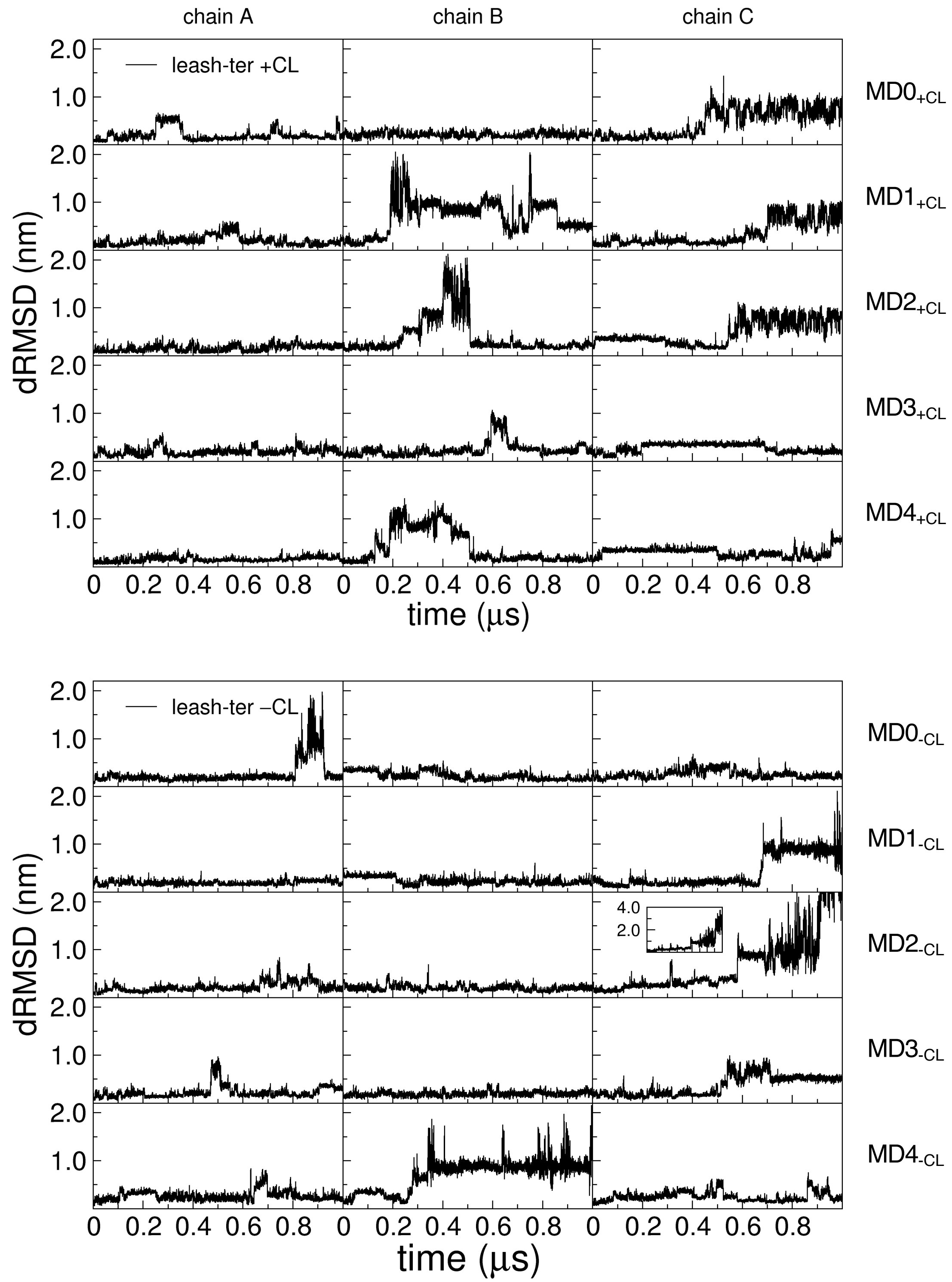

Figure S6: Same as Figure S6 for the interface between the leash-ter region (residues 94_i-98_i) and the coiled-coil core in *1mof*. The coiled-coil core interface in this case includes residues 61_i, 65_i, 69_i, 72_i, 60_{i+1}, 63_{i+1}, 64_{i+1}, 66_{i+1}, 67_{i+1}, 70_{i+1}.

Table S1: Crick parameters of the examined coiled coils[a]

TCC		R_0^b (Å)	R_1 (Å)	ω_0 (°/res)	ω_1 (°/res)	$\Delta\phi_1$ (°)	RMSD (Å)
heptad with Asn@*d*							
2wpy	X-ray	6.05	2.30	-4.2	103.5	-162.5 (*a*)	0.12
	MD+Cl^-	6.24 (.01)	2.28	-3.6	103.0	-163.3/-161.8/-161.1	0.07
	MD−Cl^-	6.07 (.02)	2.28	-3.6	102.8	-162.4/-161.6/-164.0	0.09
cubic	MD+Cl^-	6.24 (.02)	2.27	-3.6	103.1	X-163.1/-161.8/-161.1	0.10
	MD−Cl^-	6.08 (.03)	2.28	-3.6	102.9	X-162.3/-161.6/-164.0	0.12
$H18^+$	MD+Cl^-	6.18 (.01)	2.28	-3.7	103.0	-161.8/-161.9/-162.8	0.06
	MD−Cl^-	6.05 (.01)	2.28	-3.6	103.0	-161.6/-165.2/-163.0	0.10
4dzk	X-ray	6.11	2.29	-3.3	103.5	-162.5 (*a*)	0.10
	MD+Cl^-	6.32 (.01)	2.26	-3.1	103.1	-160.1/-160.3/-159.8	0.04
	MD−Cl^-	6.30 (.01)	2.26	-3.0	102.7	-158.4/-158.3/-158.1	0.04
1mof	X-ray	6.10	2.29	-4.6	105.4	-172.2 (*a*)	0.15
	MD+Cl^-	6.00 (.01)	2.25	-4.9	104.2	-165.3/-165.9/-165.4	0.14
	MD−Cl^-	6.12 (.02)	2.25	-5.0	104.3	-163.2/-164.3/-166.1	0.13
whole TCC							
2wpy	X-ray	6.31	2.29	-4.2	103.0	94.1 (*g*)	0.23
	MD+Cl^-	6.35	2.23	-3.5	103.0	91.2/93.5/92.6	0.33
	MD−Cl^-	6.29	2.22	-3.4	102.9	94.9/94.1/92.8	0.39
4dzk	X-ray	6.42	2.26	-2.8	102.8	97.3 (*g*)	0.35
	MD+Cl^-	6.49	2.23	-3.1	103.0	96.1/95.8/96.4	0.21
	MD−Cl^-	6.50	2.23	-3.1	103.0	97.0/96.5/97.0	0.22
1mof	X-ray	6.46	2.23	-3.2	103.1	89.1 (*g*)	0.75
	+Cl^-	6.34	2.17	-3.2	102.9	93.0/93.8/96.7	0.75
	−Cl^-	6.32	2.21	-3.3	103.1	90.8/91.2/89.9	0.64

[a] for the definitions of the various parameters see Fig. S8. The reported values were obtained by fitting the X-ray or the MD average structures (only the main chain is considered in the average, and the first 100 ns are discarded), the latter either with or without the bound Cl^-, using the Octave script from https://grigoryanlab.org/cccp/index.fit.php.[1] The RMSD is the root mean square distance between the ideal coiled coil and the actual structure.
[b] In the case of the superhelical radius (R_0) we report in parenthesis the associated uncertainties (for the heptad case only). The uncertainties were estimated by dividing the trajectory in three bins and taking the average structure of each bin. The three average structures were fitted and the resulting values for R_0 were used to calculate standard deviations.

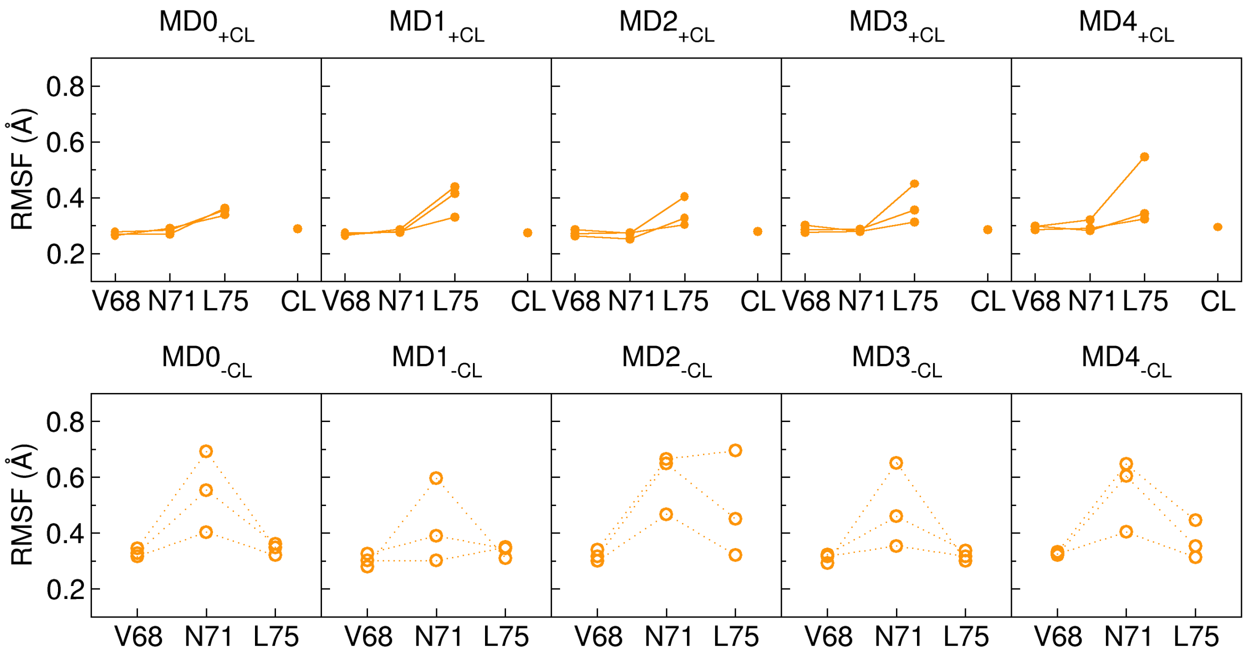

Figure S7: RMSF analysis restricted to the Asn@d and adjacent a layers for the five 1μs simulations with (top) and without (bottom) the central Cl^- ion.

Table S2: Same as Table S1 for the extra simulations of *2wpy*.

TCC		R_0 (Å)	R_1 (Å)	ω_0 ($^\circ/res$)	ω_1 ($^\circ/res$)	$\Delta\phi_1$ ($^\circ$)	RMSD (Å)
heptad with Asn@d							
ff Amber99sb-disp							
2wpy	MD+Cl^-	6.42 (.01)	2.29	-2.8	103.3	-165.6/-165.6/-165.4	0.08
	MD−Cl^-	6.14 (.02)	2.32	-3.2	102.9	-164.3/-164.9/-164.7	0.07
ff CHARMM22*							
2wpy	MD+Cl^-	6.24 (.06)	2.28	-2.9	102.9	-164.7/-164.5/-164.3	0.14
	MD−Cl^-	6.07 (.01)	2.31	-3.5	102.8	-163.9/-163.7/-164.1	0.09

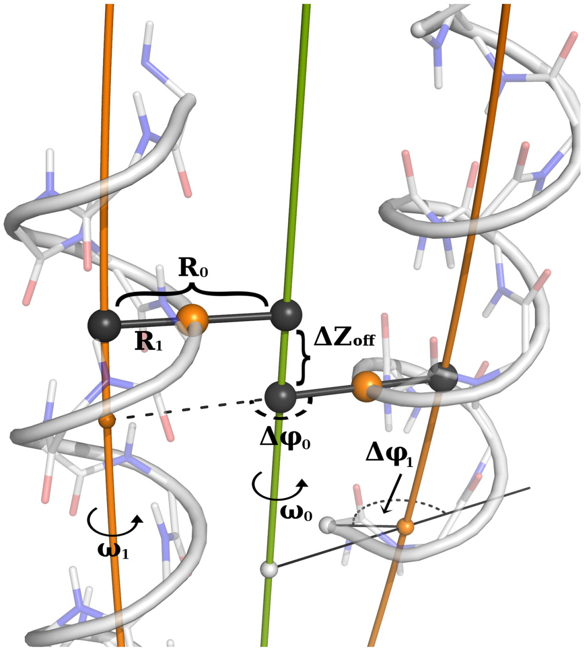

Figure S8: Visual representation of generalized Crick parameters for coiled coils. The coiled coil axis is in green, while orange curves represent the local helical axes. The gray tubes represent the helical curve through Cα atoms. $\mathbf{R_0}$ is the superhelical radius, the distance from the superhelical axis to the axis of each α-helix; $\mathbf{R_1}$ is the helical radius, the radius of an individual α-helix around its own axis; $\boldsymbol{\omega_0}$ is the superhelical frequency, indicating how many residues are required for the helix axes to complete one full 360° rotation around the superhelical axis; $\boldsymbol{\omega_1}$ is the helical frequency, the intrinsic twist of each α-helix about its own axis; $\mathbf{\Delta Z_{off}}$ is the chain axial offset between helices along the superhelical axis; $\boldsymbol{\Delta\phi_0}$ is the chain superhelical phase offset specifying each helix's angular position around the superhelical axis (120° for the regular trimeric coiled coils); $\boldsymbol{\Delta\phi_1}$ is the starting helical phase, indicating the starting heptad position of each helix. Orange balls show the inward-facing points on the helical curves corresponding to points with helical phase of π. Reproduced with permission from ref.[1] Copyright 2010 Elsevier.

Table S3: R_0 (in Å) across the simulations of *1mof* with and without the central Cl^-, at different time intervals.

simulation	0.1-0.4μs	0.4-0.7μs	0.7-1.0μs	0.1-1.0μs
$MD0_{+CL}$	6.002	5.988	6.002	6.001
$MD1_{+CL}$	6.019	6.002	6.023	6.020
$MD2_{+CL}$	6.067	6.087	6.041	6.058
$MD3_{+CL}$	6.016	6.026	6.049	6.027
$MD4_{+CL}$	6.047	6.031	6.052	6.048
$MD0_{-CL}$	6.105	6.124	6.145	6.118
$MD1_{-CL}$	6.098	6.113	6.084	6.093
$MD2_{-CL}$	6.049	6.046	6.023	6.041
$MD3_{-CL}$	6.096	6.104	6.114	6.102
$MD4_{-CL}$	6.073	6.111	6.098	6.078

Figure S9 *(following page)*: Monitoring of Cl^- unbinding event during a biased MD simulation of *2wpy* at 397 K. The time evolution of the collective variables over the full simulation is shown in the top panel. The thick lines are running averages (1ns-window for top panel and 100ps-window for middle panel). In addition to the two CVs biased during the replica-exchange metadynamics simulation – namely, the coordination number between the Asn19 side chains and Cl, and the distance RMSD (dRMSD) of Cα atoms at the coiled-coil interface – the RMSD of inter-chain distances between Asn19 side-chain atoms is also reported, clearly showing that upon unbinding the Asn19 triad adopts a different configuration. The time window corresponding to the Cl^- unbinding event is enlarged in the middle panel. The bottom panels show representative top and side views of the system at selected times. Only residues and water molecules/ions within the binding site or in the immediate vicinity of the central chloride ion (shown as a larger green sphere) are displayed.

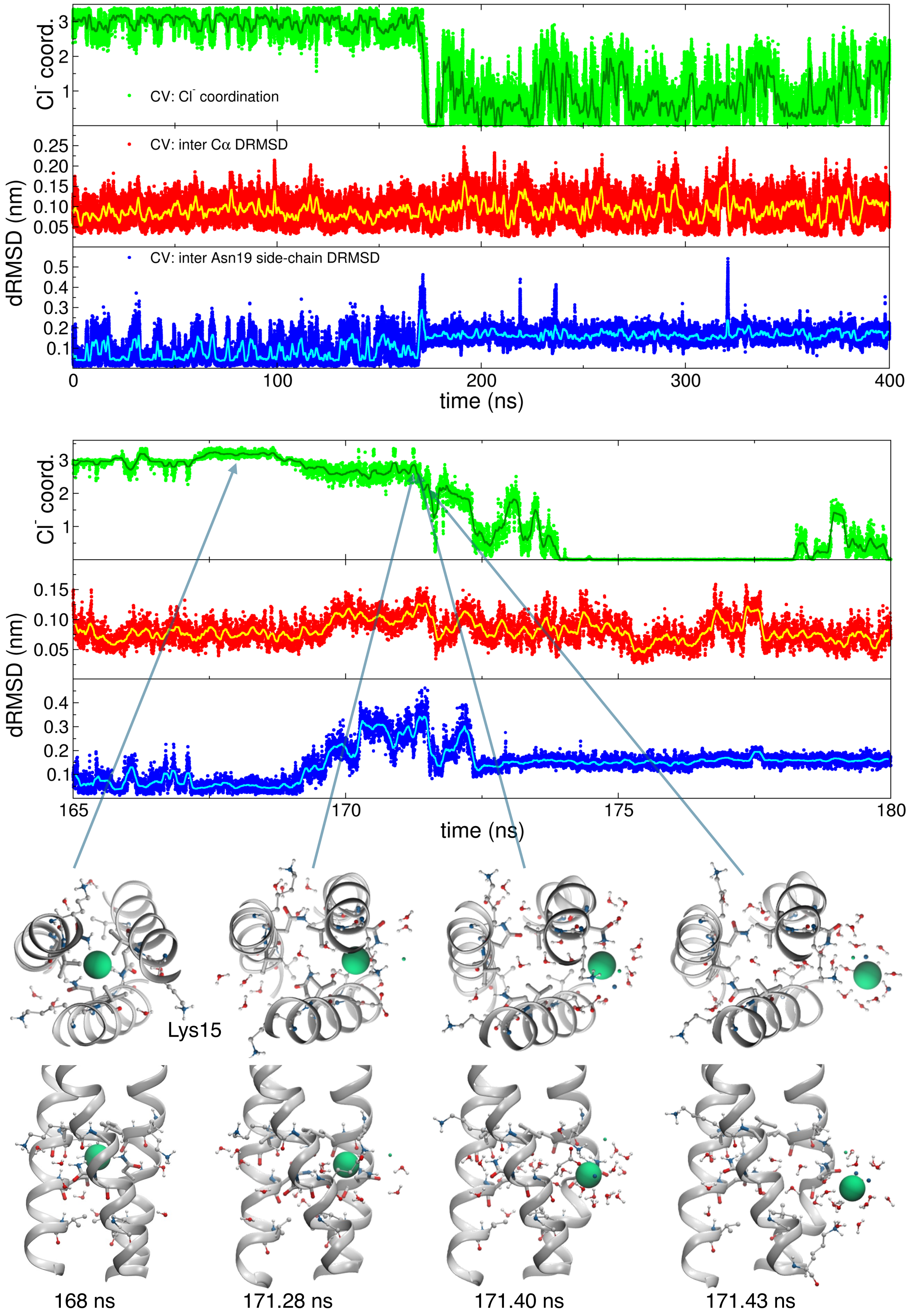

Cl⁻ coord.
CV: Cl⁻ coordination
CV: inter Cα DRMSD
CV: inter Asn19 side-chain DRMSD
dRMSD (nm)
time (ns)
Lys15
168 ns
171.28 ns
171.40 ns
171.43 ns

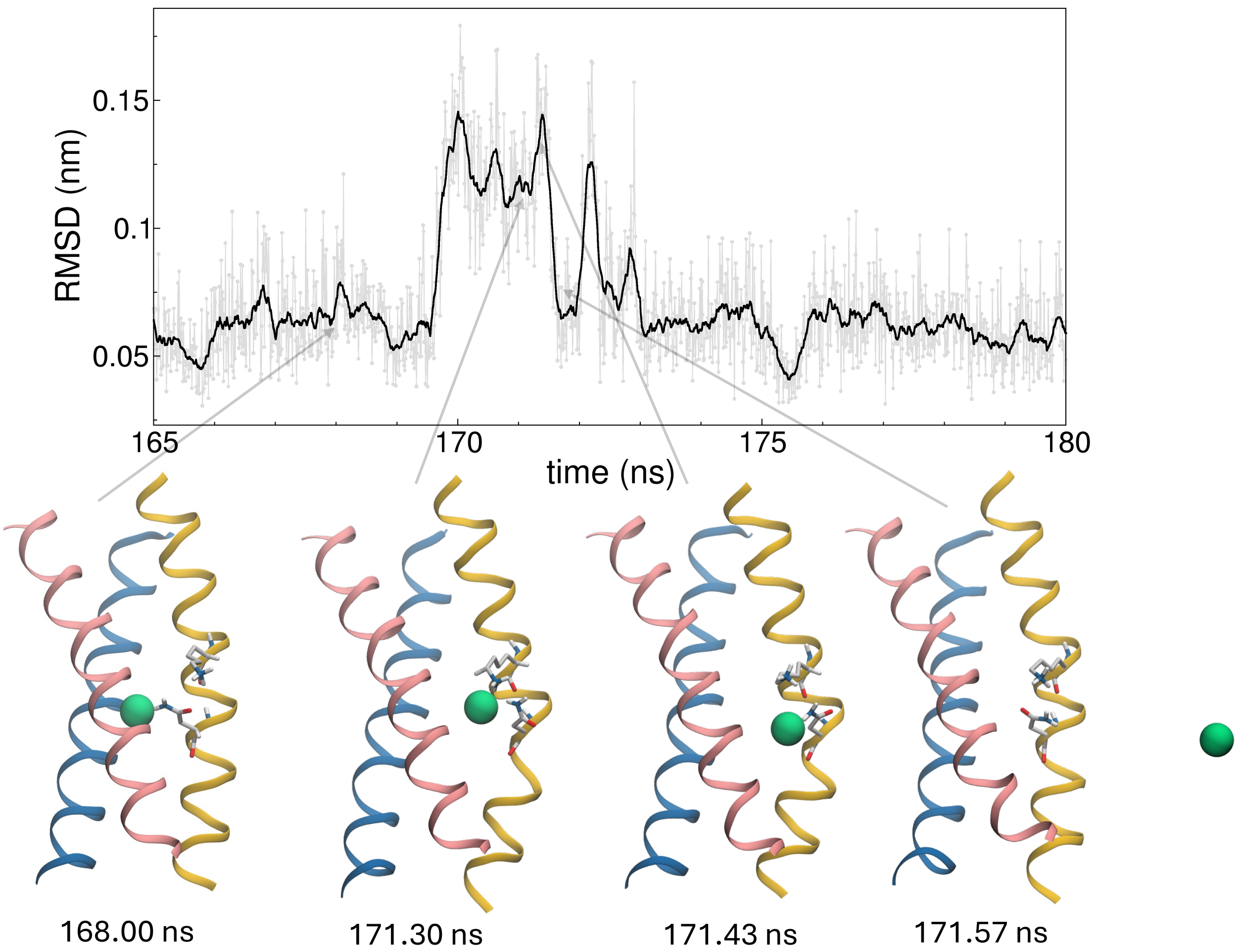

Figure S10: α-helix distortion during the unbinding event. Top: RMSD of backbone atoms of α-helix B (the one depicted in yellow in the representation below) with respect to the X-ray structure in the same simulation of Figure S9. The thicker black line is a 100ps running average. Bottom: representative views at selected times (Lys15 and Asn19 of the helix undergoing the distortion are shown).

Table S4: ΔG values[a] for each $Cl \rightarrow 0$ or $0 \rightarrow Cl$ run of *2wpy*

	$\Delta G^{Coul}_{Cl\rightarrow 0}$	$\Delta G^{vdW}_{Cl\rightarrow 0}$	ΔG^{restr}_{prot}	$\Delta G_{Cl\rightarrow 0}$	ΔG_{bind}
			2wpy cubic		
A[b]	393.9 (4.2)	-19.7 (1.2)	2.7 (0.1)	355.5 (4.8)	30.0 (4.9)
	394.0 (2.1)	*-19.8 (0.6)*	*2.7 (0.1)*	*355.6 (2.5)*	*29.9 (2.6)*
A'	394.4 (2.2)	-19.1 (0.9)	2.7 (0.3)	356.7 (2.1)	28.8 (2.2)
	394.5 (1.1)	*-19.2 (0.5)*	*2.7 (0.1)*	*356.8 (1.0)*	*28.7 (1.1)*
A"	395.5 (2.5)	-19.1 (0.5)	3.1 (0.1)	358.2 (2.7)	27.3 (2.8)
	395.6 (1.2)	*-19.1 (0.3)*	*3.0 (0.1)*	*358.2 (1.3)*	*27.3 (1.4)*
B	391.1 (2.0)	-20.2 (0.8)	2.8 (0.2)	352.5 (2.4)	33.0 (2.5)
	391.3 (0.9)	*-20.3 (0.4)*	*2.8 (0.1)*	*352.6 (1.2)*	*32.9 (1.3)*
			2wpy tr. oct. ($Cl \rightarrow 0$)		
A	394.1 (2.0)	-22.6 (0.7)	3.0 (0.1)	353.2 (1.1)	32.3 (1.2)
	394.3 (0.9)	*-22.7 (0.3)*	*3.0 (0.1)*	*353.3 (0.8)*	*32.2 (0.9)*
A'	392.7 (1.4)	-20.8 (1.0)	2.7 (0.1)	353.4 (2.3)	32.1 (2.4)
	392.9 (0.7)	*-20.9 (0.5)*	*2.7 (0.0)*	*353.4 (1.0)*	*32.1 (1.1)*
B	394.4 (2.4)	-22.2 (0.5)	2.7 (0.1)	353.6 (2.8)	31.9 (2.9)
	394.6 (1.4)	*-22.2 (0.2)*	*2.7 (0.0)*	*353.8 (1.5)*	*31.7 (1.6)*
			2wpy tr. oct. ($0 \rightarrow Cl$)		
A	394.1 (1.1)	-24.0 (1.1)	4.1 (0.3)	352.9 (1.4)	32.6 (1.5)
	394.2 (0.8)	*-24.0 (0.5)*	*4.0 (0.1)*	*353.0 (0.7)*	*32.5 (0.8)*
A'	395.9 (0.4)	-24.9 (0.6)	3.4 (0.1)	353.1 (0.8)	32.4 (0.9)
	396.1 (0.5)	*-25.0 (0.3)*	*3.4 (0.1)*	*353.2 (0.4)*	*32.3 (0.5)*
B	393.5 (1.3)	-23.8 (1.1)	3.4 (0.2)	351.8 (0.7)	33.7 (0.8)
	393.6 (0.8)	*-23.8 (0.6)*	*3.4 (0.1)*	*351.8 (0.4)*	*33.7 (0.5)*
			2wpy tr. oct. $H18^+$		
A	399.8 (1.5)	-23.6 (0.9)	3.4 (0.2)	358.3 (2.3)	27.2 (2.4)
	400.0 (0.8)	*-23.6 (0.4)*	*3.4 (0.1)*	*358.5 (1.2)*	*27.0 (1.3)*
A'	404.4 (1.8)	-26.2 (0.6)	3.2 (0.1)	360.2 (2.0)	25.3 (2.1)
	404.5 (0.9)	*-26.2 (0.3)*	*3.2 (0.1)*	*360.1 (1.0)*	*25.4 (1.1)*
B	405.2 (1.8)	-24.3 (0.5)	3.2 (0.0)	362.8 (2.3)	22.7 (2.4)
	405.4 (0.9)	*-24.4 (0.3)*	*3.2 (0.0)*	*362.9 (1.1)*	*22.6 (1.2)*
			2wpy tr. oct. (ff:a14sb)		
A	360.4 (1.4)	-13.5 (1.2)	4.6 (0.4)	330.2 (1.0)	42.7 (1.1)
	360.2 (0.7)	*-13.5 (0.6)*	*4.5 (0.2)*	*329.9 (0.6)*	*43.0 (0.7)*
A'	361.5 (0.8)	-14.3 (0.8)	4.4 (0.5)	330.2 (1.3)	42.7 (1.4)
	361.2 (0.4)	*-14.2 (0.4)*	*4.2 (0.2)*	*330.0 (0.5)*	*42.9 (0.6)*
			2wpy tr. oct. (ff:a99sb-disp)		
A	377.2 (1.9)	-19.2 (0.5)	5.8 (0.4)	342.5 (2.3)	32.0 (2.4)
	377.2 (1.1)	*-19.5 (0.3)*	*5.7 (0.2)*	*342.2 (1.1)*	*32.3 (1.2)*
B	378.5 (0.8)	-17.8 (2.3)	4.8 (0.5)	344.2 (2.2)	30.3 (2.3)
	378.5 (0.4)	*-17.9 (1.2)*	*4.7 (0.2)*	*344.0 (1.1)*	*30.5 (1.2)*

[a] Values from thermodynamic integration are in roman and those from the Bennett acceptance ratio method,[2] or BAR, in italics

[b] Runs denoted with "A" start from the plain MD structures (with the ' indicating different starting velocities sets) while those denoted with "B" start from the structures at the end of run "A".

Table S5: ΔG values for the $Cl \rightarrow 0$ and $0 \rightarrow Cl$ runs of *4dzk* and *1mof*. See caption of Table S4 for details.

run	$\Delta G^{Coul}_{Cl\rightarrow 0}$	$\Delta G^{vdW}_{Cl\rightarrow 0}$	ΔG^{restr}_{prot}	$\Delta G_{Cl\rightarrow 0}$	ΔG_{bind}
			4dzk tr. oct.		
A	402.3 (2.2)	-16.9 (0.7)	3.6 (0.0)	367.6 (1.8)	17.9 (1.9)
	402.5 (1.1)	*-16.9 (0.4)*	*3.6 (0.0)*	*367.6 (0.9)*	*17.9 (1.0)*
A'	402.4 (0.8)	-16.6 (0.6)	3.7 (0.1)	367.9 (1.2)	17.6 (1.3)
	402.5 (0.4)	*-16.6 (0.3)*	*3.6 (0.0)*	*368.0 (0.6)*	*17.5 (0.7)*
			4dzk-cap tr. oct.		
A	401.9 (1.1)	-16.7 (0.5)	3.8 (0.2)	367.5 (1.6)	18.0 (1.7)
	402.1 (0.5)	*-16.8 (0.2)*	*3.8 (0.1)*	*367.5 (0.8)*	*18.0 (0.9)*
B	401.5 (1.8)	-16.8 (0.8)	3.8 (0.1)	367.0 (1.3)	18.5 (1.4)
	401.6 (0.9)	*-16.8 (0.4)*	*3.8 (0.1)*	*367.1 (0.7)*	*18.4 (0.8)*
			1mof tr. oct.		
A	445.7 (0.8)	-9.9 (1.6)	1.9 (0.2)	416.4 (1.6)	-30.9 (1.7)
	445.8 (0.4)	*-9.9 (0.8)*	*1.9 (0.1)*	*416.5 (0.8)*	*-31.0 (0.9)*
A'	446.5 (1.0)	-9.9 (0.6)	1.9 (0.2)	417.3 (0.9)	-31.8 (1.0)
	446.9 (0.5)	*-10.1 (0.3)*	*1.9 (0.1)*	*417.4 (0.4)*	*-31.9 (0.5)*
B	443.8 (1.0)	-13.7 (1.3)	2.2 (0.1)	411.0 (1.9)	-25.5 (2.0)
	444.1 (0.5)	*-14.2 (0.8)*	*2.2 (0.1)*	*410.8 (1.1)*	*-25.3 (1.2)*
			1mof tr. oct. $(0 \rightarrow Cl)$		
A	437.2 (1.3)	-9.0 (0.8)	2.1 (0.1)	409.0 (1.5)	-23.5 (1.6)
	437.5 (0.7)	*-9.2 (0.4)*	*2.1 (0.0)*	*409.1 (0.8)*	*-23.6 (0.9)*
B	442.4 (0.5)	-9.3 (0.5)	2.3 (0.1)	414.1 (1.0)	-28.6 (1.1)
	442.6 (0.3)	*-9.3 (0.2)*	*2.3 (0.0)*	*414.2 (0.5)*	*-28.7 (0.6)*
			1mof tr. oct. $(Cl \rightarrow 0)$ open leash[a]		
A	430.0 (2.8)	-18.8 (2.9)	2.8 (0.0)	392.8 (5.2)	-7.3 (5.3)
	430.1 (1.4)	*-18.8 (1.4)*	*2.9 (0.0)*	*392.9 (2.6)*	*-7.4 (2.7)*
B	429.6 (1.6)	-16.9 (1.7)	2.9 (0.0)	394.3 (1.8)	-8.8 (1.9)
	429.7 (0.8)	*-16.9 (0.8)*	*2.9 (0.0)*	*394.4 (0.9)*	*-8.9 (1.0)*

[a] the starting structure for run A of this set is the final structure of $MD2_{-CL}$, with an "open" leash. The internal water was replaced by a Cl^-, and a short MD run (10ns) was performed for equilibration before the alchemical run.

Table S6: ΔG values for the $Cl \rightarrow W$ transformation for *1mof*

run	$\Delta G^{Coul}_{Cl\rightarrow W}$	$\Delta G^{vdW}_{Cl\rightarrow W}$	$\Delta G_{Cl\rightarrow W}$	ΔG^{a}_{bind}
		1mof tr. oct.		
A	449.9 (1.4)	-55.3 (0.3)	394.6 (1.4)	-26.6 (2.0)
	450.2 (0.7)	*-55.1 (0.2)*	*395.1 (0.7)*	*-25.7 (1.7)*
A'	450.8 (2.4)	-55.6 (0.7)	395.2 (2.9)	-27.2 (3.2)
	451.0 (1.1)	*-55.4 (0.3)*	*395.7 (1.4)*	*-27.7 (2.0)*
B	445.7 (3.0)	-56.4 (0.3)	389.4 (3.1)	-21.4 (3.4)
	446.5 (1.4)	*-56.2 (0.1)*	*390.3 (1.5)*	*-20.9 (1.5)*
		water		
	484.1 (1.2)	-126.1 (0.7)	358.0 (1.4)	-
	484.2 (0.2)	*-124.8 (0.1)*	*359.4 (0.2)*	-

[a] $\Delta G_{bind} = \Delta G^{water}_{Cl\rightarrow W} - \Delta G^{protein}_{Cl\rightarrow W} + RT \ln [H_2O]/c_0$ where the last term is 9.95 kJ/mol.
